# A Deep Learning Framework for the Detection of Abnormality in Cerebral Blood Flow Velocity Using Transcranial Doppler Ultrasound

**DOI:** 10.3390/diagnostics13122000

**Published:** 2023-06-08

**Authors:** Naima Nasrin Nisha, Kanchon Kanti Podder, Muhammad E. H. Chowdhury, Mamun Rabbani, Md. Sharjis Ibne Wadud, Somaya Al-Maadeed, Sakib Mahmud, Amith Khandakar, Susu M. Zughaier

**Affiliations:** 1 Department of Biomedical Physics & Technology, University of Dhaka, Dhaka 1000, Bangladeshkanchon.k.podder@bmpt.du.ac.bd (K.K.P.);; 2 Department of Electrical Engineering, Qatar University, Doha 2713, Qatar; 3 Department of Computer Science and Engineering, Qatar University, Doha 2713, Qatar; 4 Department of Basic Medical Sciences, College of Medicine, Qatar University, Doha 2713, Qatar

**Keywords:** transcranial doppler ultrasound, middle cerebral artery, Self-ONN, signal classification

## Abstract

Transcranial doppler (TCD) ultrasound is a non-invasive imaging technique that can be used for continuous monitoring of blood flow in the brain through the major cerebral arteries by calculating the cerebral blood flow velocity (CBFV). Since the brain requires a consistent supply of blood to function properly and meet its metabolic demand, a change in CBVF can be an indication of neurological diseases. Depending on the severity of the disease, the symptoms may appear immediately or may appear weeks later. For the early detection of neurological diseases, a classification model is proposed in this study, with the ability to distinguish healthy subjects from critically ill subjects. The TCD ultrasound database used in this study contains signals from the middle cerebral artery (MCA) of 6 healthy subjects and 12 subjects with known neurocritical diseases. The classification model works based on the maximal blood flow velocity waveforms extracted from the TCD ultrasound. Since the signal quality of the recorded TCD ultrasound is highly dependent on the operator’s skillset, a noisy and corrupted signal can exist and can add biases to the classifier. Therefore, a deep learning classifier, trained on a curated and clean biomedical signal can reliably detect neurological diseases. For signal classification, this study proposes a Self-organized Operational Neural Network (Self-ONN)-based deep learning model Self-ResAttentioNet18, which achieves classification accuracy of 96.05% with precision, recall, f1 score, and specificity of 96.06%, 96.05%, 96.06%, and 96.09%, respectively. With an area under the ROC curve of 0.99, the model proves its feasibility to confidently classify middle cerebral artery (MCA) waveforms in near real-time.

## 1. Introduction

Non-invasive disease detection techniques are considered to be more convenient compared to invasive diagnostic procedures. Ultrasound is one of the most common non-invasive imaging techniques, and owing to its efficacy, low cost, and zero radiation hazard, it is considered the safest imaging modality [1]. The most common uses of diagnostic ultrasound involve fetal heart monitoring in pregnant women and observation of abdominal organs and heart valves. Pulsed doppler ultrasound is an imaging technique where short bursts of ultrasonic waves are applied to the insonation area for imaging the movements of the respective organs, especially for the measurement of blood flow through the blood vessels [2]. Owing to the brain’s inability to store energy, consistent blood flow is essential for keeping up with the metabolic demands of the brain and supporting cerebral function [3,4]. This implies that an alteration in cerebral blood flow velocity can be an indication of a disruption in the systemic flow caused by any neurological disorder [5].

Doppler ultrasound is a low-cost, non-invasive diagnostic tool commonly used to diagnose a wide spectrum of neurocritical disorders [6,7]. Primarily, the diagnosis is made by measuring the change in blood flow velocity [7,8,9]. Transcranial doppler (TCD) ultrasonography, which operates on the same concept as doppler ultrasound, can be a useful indication for diagnosing neurological diseases such as artery stenosis and occlusion in patients by observing a change in the blood flow velocity profile in the relevant arteries. For TCD scans, the basal arteries are used to quantify blood flow [10].

### 1.1. Related Work

Transcranial doppler ultrasound (TCD) is a non-invasive tool widely used for the detection of neurological diseases [7,10]. Using a high-frequency ultrasound signal, blood flow velocities in the major basal arteries, including the middle cerebral artery (MCA) and internal carotid artery (ICA), are measured. In recent years, the potentials of deep learning and neural networks are being explored for the efficient detection of arterial diseases from doppler spectrograms. Übeyli and Güler developed a technique for detecting abnormalities in the ophthalmic artery (OA) and the internal carotid artery (ICA) by decomposing the signals using wavelet transform to express them in time–frequency format [11]. Using the Levenberg–Marquardt (LM) optimization technique on a Multilayer Perceptron (MLP) network, they achieved classification accuracies of 95.52% and 97% for OA and ICA disorder, respectively [11]. Seddik and Shawky [12] described a cost-effective screening approach for carotid artery disorders. They extracted different signal features from the frequency spectrogram after pre-processing for noise elimination. The classification accuracies achieved for normal and occlusion patterns were 91.67% and 95.85%, respectively, using the MLP classifier [12]. Wavelet transform-based spectral analysis of the doppler signal from ICA was conducted in [13] using a technique similar to [11]. An MLP was trained with LM optimization for stenosis and occlusion detection. The model achieved accuracies of 96%, 96.15%, and 96.30% for healthy subjects, subjects with arterial stenosis, and subjects with blood vessel occlusion, respectively [13]. An intima–media thickness-based plaque identification technique for early detection of stroke was reported by [14]. They trained an MLBPN with LM optimization to classify ultrasound images of the carotid artery and achieved an accuracy of 89.43%. Uğuz proposed a classification algorithm based on the Learning Vector Quantization Neural Network (LVQ NN) for classifying ICA doppler data [15]. The Burg autoregressive spectrum was utilized to derive power spectral density (PSD) characteristics. LVQ NN achieved a classification accuracy of 97.91% using the five-fold cross-validation method.

One of the issues with the existing literature on the topic is that the majority of the literature focuses on doppler ultrasound taken from the carotid artery. Since cerebral arteries transport blood to deeper parts of the brain, ultrasound pictures of the middle cerebral artery have significant clinical significance [4]. Mei et al. [16] provide a similar study in which they evaluated TCD images captured from the middle cerebral artery. The CNN VGG16 model was utilized to classify the images into the stenosis and non-stenosis groups. They reported a classification accuracy of 80%, with a sensitivity of 84% and a specificity of 86%. Individual and Recurrent Neural Networks (RNNs) were utilized to classify the MCA doppler signal and data from other basal arteries in [17]. However, the model achieved accuracy ranging between only 71.1% and 75.89% for MCA ultrasound signals. Li et al. created a neural network-based system for detecting arterial stenosis [18]. They have utilized a synthetic peripheral pulse wave-based dataset that represents varying degrees of vascular stenosis. The disadvantage of their model, however, was that it was greatly dependent on stenosis severity. For less severe stenosis, the model’s accuracy is weak, rendering it unreliable for early diagnosis of stenosis.

### 1.2. Motivation

TCD signals are typically corrupted with speckle noise and motion artefacts since the quality of the signal depends heavily on the skill set of the operator [8,19]. Therefore, signal reconstruction techniques [20] or other noise reduction methods [21,22] can be employed to enhance the signal quality. For this reason, the successful classification of a variation in blood flow velocity requires the utilization of a signal-processing method that is both efficient and reliable. Owing to the efficiency and reliability of computer-aided detection and classification techniques, these are preferred over other detection techniques [23].

The unique morphologies of TCD waveform acquired from different subjects can be a significant indication of the presence of any neurological conditions [8]. This justifies our approach of categorizing TCD waveform being a classification problem. Biomedical signal classification using a decision-based algorithm [24,25] and deep learning [26,27,28] approach is gaining the attention of researchers for various tasks. Recent studies have proposed several approaches to address the TCD waveform classification problem [23,29,30,31]. However, the problem domain is still a work in progress due to the lack of comprehensiveness of the results. The use of neural networks is a significant method for signal processing, especially when it comes to the classification of TCD ultrasound waveforms [11,31,32,33].

By employing a deep learning model, signal classification can be made easy and reliable and be used in a clinical setting. With this in mind, we have designed a study that will contribute to the medical field as follows:

- A novel TCD ultrasound waveform classification system that incorporates cerebral blood flow velocity (CBFV) waveform estimation, data cleaning and segmentation, and classification for classifying data from doppler ultrasounds in both healthy subjects and intensive care unit subjects.
- Two novel SelfONN architectures, (a) a 1D version of ResNet18 and (b) a 1D version of ResNet architecture with a multiheaded attention layer, have been proposed for 1D binary classification, where TCD ultrasound can be used to identify ICU (in this study, “ICU” is an umbrella term used for representing MCA waveforms from hydrocephalus, traumatic brain injury, and intraparenchymal or subarachnoid hemorrhage patients) patients from healthy subjects by analyzing the maximal cerebral blood flow velocity (CBFV) waveforms.

This paper is organized into five sections and the rest of the paper is organized as follows. Section 2 elaborates on the methods and materials used along with the experimental setup. The experimental results and discussion are presented in Section 3. Section 4 presents the concluding remarks and finally, Section 5 discusses the limitations and future scopes of this study.

## 2. Materials and Methods

**Figure 1:**
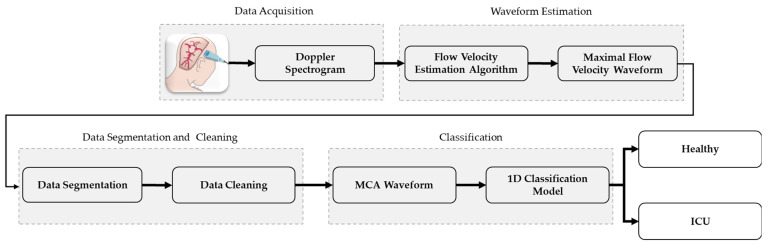
A graphical representation of a novel TCD ultrasound waveform classification system.

This study uses a range of different techniques for data pre-processing and classification of healthy subject and ICU patient data. This section presents a detailed discussion of the step-by-step procedure followed during the study. The overall procedure is summarized in Figure 1. The data-acquisition process is completed in the literature [9] and the other processes are investigated in this study.

### 2.1. Overview of the Dataset

This study uses a transcranial doppler (TCD) ultrasound dataset collected from IEEE Dataport (https://ieee-dataport.org/open-access/transcranial-doppler-ultrasound-database-philips-cx50-ultrasound-system, accessed on 3 April 2023), which was provided by Wadehn et al. [9]. The dataset comprises healthy subject data as well as data from patients with known neurocritical disorders. Table 1 contains the overview of the dataset, and the detailed protocols for clinical data collection can be found in [9,34].

### 2.2. Signal Extraction

**Figure 2:**
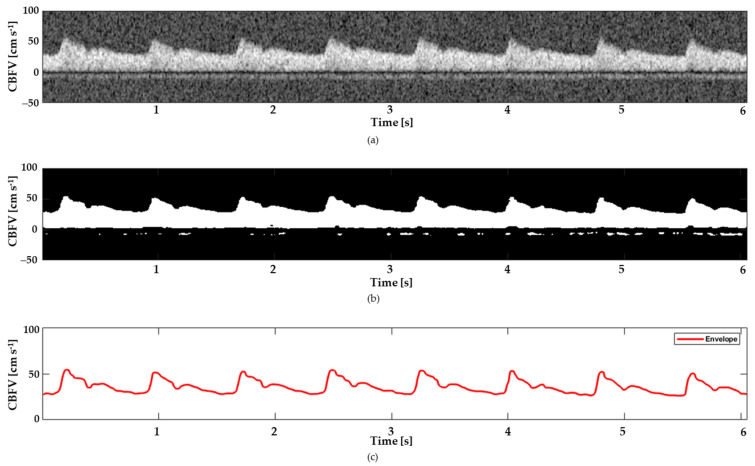
The outputs of the signal extraction process in the top–down view: (**a**) grayscale TCD spectrogram, (**b**) black-and-white spectrogram, and (**c**) envelope signal.

To acquire the transcranial doppler (TCD) ultrasound signals, the segments of the MCA were insonated with short pulses of ultrasound waves with a carrier frequency of 1.75 MHz. The ultrasound pulses were reflected from the moving red blood cells and captured by the transducer as TCD echo signals. Wadehn et al. proposed a flow velocity estimation algorithm that takes the TCD ultrasound signals and returns a 1D maximal flow velocity waveform [9]. Using STFT (short-time Fourier transform), envelope tracking, and some post-processing, the TCD spectrogram was transformed into a 2D spectrogram. A black-and-white image of the spectrogram was created using an envelope tracing algorithm also developed by the same author. When the spectrogram was binarized, the speckle noise was eliminated using a 2D median filter with a kernel size of 0.03 s (horizontally) and 5 cm s^−1^ (vertically). The maximal flow velocity envelope was detected via an adaptive threshold method due to the overlap between the signal-carrying portion and the noise of the black-and-white spectrogram [35]. The maximal flow velocity envelope is traced on a time sample basis employing two physiological sanity checks, which were determined from the signal quality index. Different stages of signal extraction are shown in Figure 2.

### 2.3. Manual Data Annotation

In this investigation, we used the technique provided in [9] to extract MCA doppler ultrasound signals from TCD ultrasound images. Several artefacts and signal cuts of varying lengths were found in the retrieved data. After removing the signal segments containing signal cuts, the remaining signals were segmented in 1024 segment lengths at a sampling frequency of 217 Hz.

**Figure 3:**
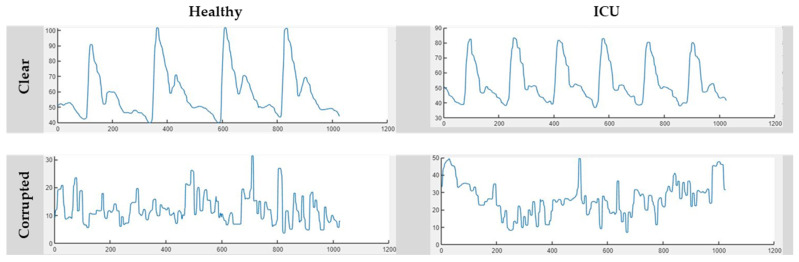
Representative segments of clear and corrupted signals across the Healthy and ICU classes.

**Figure 4:**
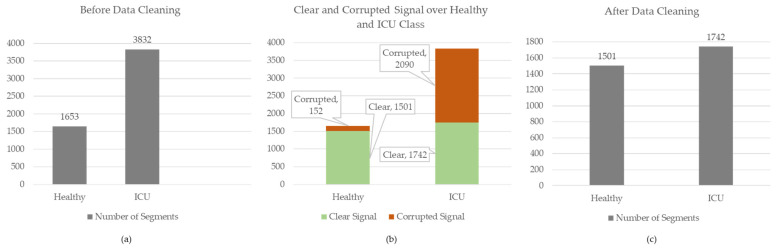
Details of data segmentation before and after data cleaning. From left to right, (**a**) a graphical representation of the number of segments across binary classes before data cleaning, (**b**) the graphical representation of clear and corrupted data numbers across the classes, and (**c**) a chart view of the number of the segments in Healthy and ICU classes used in this study.

Afterwards, these segments were manually annotated by human supervision. The segments were annotated into two categories, clear segments and noisy segments, and Figure 3 represents four samples from the Healthy and ICU classes. The signal portions that have no visible artefacts or distortions were categorized as *Clear Signals*. Some corrupted segments with NaN values, plane lines, or no sign of physiological information were eliminated from the dataset during manual annotation. From the remaining signal portions, the cleanest or negligible distortions were labelled as *Corrupted Signals*. A total of 5468 segments were created from the Healthy and ICU classes during the segmentation process with 1024 segment lengths. For the Healthy class, the numbers of clear and corrupted segments were 1501 and 152, respectively, whereas they were 1742 and 2090 for the ICU class, respectively. Details of dataset segmentation and labelling can be seen in Figure 4.

### 2.4. Model Architecture

For the classification of signals into the two categories, “Healthy” and “ICU patients”, two Self-Organizing Operational Neural Network (Self-ONN)-based novel 1D classification models were proposed. The details about Self-ONN and the architecture of the 1D classification models are described in the following subsections.

#### 2.4.1. Self-ONN

To surpass the drawbacks of Convolutional Neural Networks (CNNs), Operational Neural Networks (ONNs) were proposed in [36]. Conventional CNNs function through linear convolutional operators in their neurons and layers for feature learning. According to Equation (1) [36], the output of the $k^{th}$ neuron in the $l^{th}$ layer of an one-dimensional CNN can be expressed as:

(1) $$x_{k}^{l}=b_{k}^{l}+\sum_{i=0}^{N_{l-1}}x_{ik}^{l}$$

where, $b_{k}^{l}$ is the bias associated with the neuron and $x_{ik}^{l}$ is the $l^{th}$ layer’s $k^{th}$ neuron output, which can be further expressed as Equation (2):

(2) $$x_{ik}^{l}(m)=Conv1D\left(w_{ik},y_{i}^{l-1}\right)\equiv \sum_{r=0}^{k-1}w_{ik}^{l}(r)y_{i}^{l-1}(m+r)$$

where, $w_{ik}^{l}$ represents the weight of the kernel connecting the $i^{th}$ neuron of the ${\left(l-1\right)}^{th}$ layer to the $k^{th}$ neuron of the $l^{th}$ layer, $y_{i}^{l-1}$ represents the ${\left(l-1\right)}^{th}$ layer’s $i^{th}$ neuron output, and ‘*m*’ and ‘*r*’ are convolutional operators. ONNs can address the homogeneity issues of CNNs by employing heterogeneous neurons through non-linear convolutional operators which can learn complex model functions with minimal network complexity [37]. As reflected in Equation (3), every single neuron in the ONNs can be assigned unique nodal (ψ) and pool (P) operators.

(3) $$\bar{x_{ik}^{l}}(m)={P_{k}^{l}\left(\psi_{k}^{l}\left(w_{ik}^{l}(r){,y}_{i}^{l-1}(m+r)\right)\right)}_{r=0}^{k-1}$$

Regardless of having non-linear operators, early ONNs were rigid due to dependence on a bunch of pre-set operators by the user. To solve this challenge, Kiranyaz et al. [38] proposed Self-Organizing ONNs or Self-ONNs in short, which can automatically determine the best set of problem-specific non-linear operators during training. Self-ONN uses Taylor series approximation for the non-linear transformation of each generative neuron to reach an even higher level of diversity and flexibility. Based on [38,39,40], the contribution of the $i^{th}$ neuron in generating the feature map $x_{ik}^{l}$ from the ${\left(l-1\right)}^{th}$ layer to the $l^{th}$ layer of a Self-ONN model can be expressed by Equation (4) as follows:

(4) $$\tilde{x_{ik}^{l}}\left(m\right)=\sum_{r=0}^{K-1}\sum_{q=1}^{Q}\tilde{\psi_{k}^{l}}\left(w_{ik}^{l\left(Q\right)}\left(r\right),y_{i}^{l-1}\left(m+r\right)\right)\;=\sum_{r=0}^{K-1}\sum_{q=1}^{Q}w_{ik}^{l\left(Q\right)}\left(r,q\right){\left(y_{i}^{l-1}\left(m+r\right)\right)}^{q}\equiv \sum_{q=1}^{Q}Conv1D\left(w_{ik}^{l\left(Q\right)},{\left(y_{i}^{l-1}\right)}^{q}\right)$$

where, $w_{ik}^{l\left(Q\right)}$ is the *K* × *Q* dimensional kernel matrix between the *i^th^* neuron from the ${\left(l-1\right)}^{th}$ layer and the $k^{th}$ neuron at the $l^{th}$ layer. Here, the hyperparameter *Q* can be tweaked to control the degree of Taylor series approximation while $w_{ik}^{l\left(Q\right)}$ is the learnable kernel, unlike CNNs and ONNs. Finally, the output of a single neuron can be formulated as in Equation (5):

(5) $$\tilde{x_{k}^{l}}=b_{k}^{l}+\sum_{i=0}^{N_{l-1}}\tilde{x_{ik}^{l}}$$

Mentionable that, with the *Q* = 1 setting, a Self-ONN acts like a CNN as there is no non-linearity in the first term of the Taylor series approximation. SelfMLP, which is a variant of MLP, can also be designed from the SelfONN layer.

#### 2.4.2. Self-ResNet18

**Figure 5:**
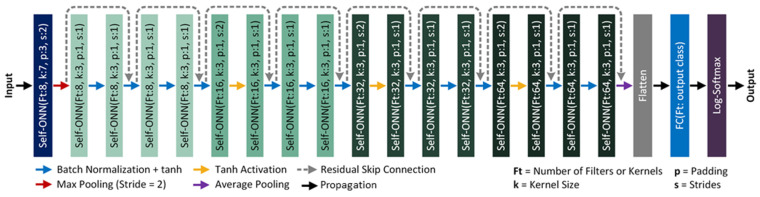
The architecture of the proposed Self-ResNet18 model. The different colors of the boxes represent different layers and residual blocks in the proposed Self-ResNet18 model.

The Self-ResNet18 model proposed in this study adopts the widely used CNN-based most lightweight version of the ResNet models proposed by He et al. [41] in 2015. Being one of the oldest deep learning architectures, ResNet models have been used in many studies to solve various types of problems. In this study, we replace the CNN layers of the ResNet18 model with the Self-ONN layers to construct the architecture of the proposed Self-ResNet18. The architecture of Self-ResNet18 has been drawn in Figure 5.

Our proposed Self-ResNet18 model developed in PyTorch contains 18 Self-ONN layers with *Q* = 3, as depicted in Figure 5. We started with 8 kernels or filters in the initial layer, which was doubled up after each Self-ONN layer with a stride of two. Based on the original implementation strategies [38], each Self-ONN block was followed by a “batch normalization” layer and a “tanh” activation function. The batch normalization layer was avoided during the down-sampling process. After the last residual block, which contained 64 filters, we implemented an average-pooling layer followed by a flattening layer to flatten the features into a single array. Then we passed the feature vectors through a fully connected (FC) dense layer that performed a linear transformation of the incoming features using a linear activation function [42]. This multi-layered perceptron (MLP)-based dense layer contained filters equal to the number of output classes (which is two in this case, “Healthy” and “ICU patients”, making it a binary classification problem) to aid the classification process based on refined features from previous layers. Finally, we implemented a “Log-Softmax” layer [43] as the final activation function to help in the classification process.

#### 2.4.3. Self-ResAttentioNet18

**Figure 6:**
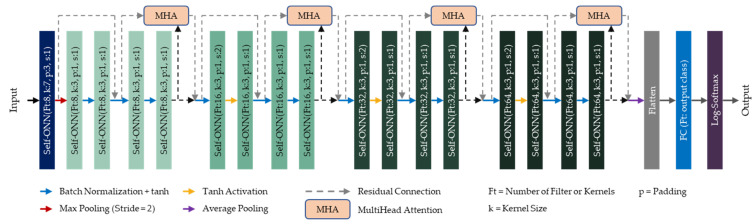
Model architecture of Self-ResAttentioNet18. The different colors of the boxes represent different layers and residual blocks in the proposed Self-ResAttentioNet18 model.

This study proposes an attention-based version of the Self-ResNet18 model, which is called the Self-ResAttentioNet18. It is used to classify MCA signals acquired from subjects into “Healthy” and “ICU” categories. Figure 6 represents the illustration of Self-ResAttentioNet18, where every two SelfONN layers after the first one is a Residual Block. In this architecture, a multi-head attention layer of several heads of 2 was added to each alternative Self-Residual block. The attention layer requires query, key, and value. Every input feature of the residual block or the identity features was used as a query and value, while the output of the residual block was used as a key. The jointly attended feature was further added to the residual block output and propagated to the next block. The Self-ResAttentioNet18 is identical to the Self-ResNet18 architecture illustrated in Figure 5, with only four multi-head attention layers added to it to jointly attend to information from the identity feature and the output feature.

### 2.5. Experimental Setup

The deep learning models discussed above were investigated on the dataset provided by [9] after preprocessing. For the classification purpose, the healthy subjects were labelled as ‘Healthy’, while the subjects with hydrocephalus, traumatic brain injury, and intraparenchymal or subarachnoid hemorrhage were labelled as ‘ICU Patients’. Thus, the problem was considered a binary classification problem. Each study was conducted twice during training, validation, and testing, once with the standard SelfRes-Net18 model and once with an enhanced Self-ResAttentioNet18 model that included multiheaded-attention layers. Both models’ efficacies were evaluated by using q-values in the range [1,3,5].

To minimize bias and data scarcity, 5-fold cross-validation was employed with 80% signal overlapping. The splitting and fold creation was performed based on session IDs as shown in Table 2. A percentage of 10% of the overall training sample was moved to the validation set to validate the model learning process during training. The objective of session-wise splitting was to minimize data bias during training and validation and testing, meaning the data used for training will not be present in the test data. This method of the 5-fold training, validation, and test data splitting ensures data stratification [44].

The models used in this study use a Python 3.7 environment and several PyTorch libraries. For training and testing, Google-Colab was used along with its high-performing resources. A 16 GB Tesla T4 GPU was used for this study. For the classification of transcranial doppler (TCD) ultrasound signals, the following hyperparameters in Table 3 were used during the model training and test phases.

### 2.6. Performance Metrics

For the quantitative analysis of the classification model, several parameters were calculated. In this work, signal classification was mainly done from the waveform morphologies of Heathy and ICU subjects’ TCD scans. The performance of the proposed model was evaluated using measures such as precision, overall accuracy, recall, specificity, and f1 score, with a 90% confidence interval (CI) in addition to receiver operating characteristic (ROC) curves. Due to the difference in sample sizes between Healthy and ICU subjects, weighted recall, precision, f1 score, and specificity measures were determined. Equations (6)–(10) describe weighted precision, sensitivity or recall, specificity, *F*1 score, and overall accuracy quantitatively.

(6) $$Precision=\frac{TP}{TP+FP}$$

(7) $$Recall=\frac{TP}{TP+FN}$$

(8) $$Specificity=\frac{TN}{FP+TN}$$

(9) $$F1\;score=\frac{2\times Precision\times Recall}{Precision+Recall}$$

(10) $$Overall\;Accuracy=\frac{TP}{TP+FP+TN+FN}$$

Here, *TP* = true positive, *FP* = false positive, *TN* = true negative, and *FN* = false negative.

A receiver operating characteristic (ROC) curve is a graph representing how well a binary classifier system works as the threshold for discrimination is varied. This graph shows the true positive rate (sensitivity) against the false positive rate (1-specificity) for different threshold settings.

(11) $$Fasle\;Positive\;Rate=\frac{FN}{TP+FN}$$

The ROC curve is a useful tool for figuring out how well a binary classifier works because it shows how the true positive rate and the false positive rate work together. The AUC of a perfect classifier is 1, while the AUC of a classifier that guesses at random is 0.5. The ROC curve can be used to evaluate the performance of a classifier for imbalanced classes. The curve can also be used to find the optimum threshold for a classifier. The point on the ROC curve that is closest to the top left corner indicates the best balance between true positives and false positives.

## 3. Results and Discussion

The goal of the study is to identify the optimum model configuration for efficient classification. For evaluating the performance of the proposed models (Self-ResNet18 and Self-ResAttentioNet18), different performance metrics were calculated. The performance metrics achieved from different models are discussed in the following subsections.

### 3.1. Accuracy-Based Comparison

**Figure 7:**
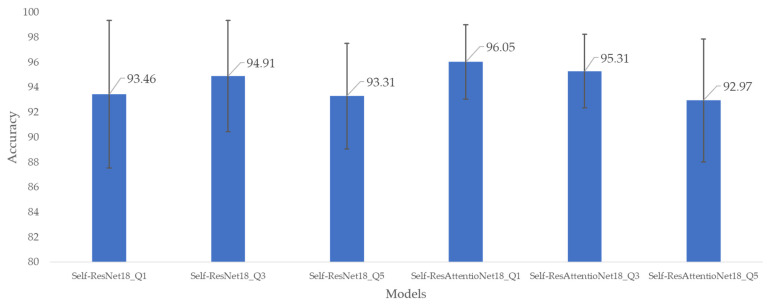
Mean and standard deviation of fold-wise accuracy of different models for the six versions of models from Self-ResNet18 and Self-ResAttentioNet18 model architecture.

The five-fold cross-validation produces classification accuracy across the five folds. For similar experimental setups, different models produce different performance metrics. The accuracy-based comparison of the proposed Self-ResNet18 model without the attention layer and Self-ResAttentioNet18 with the attention layer is given in Figure 7. The best accuracy achieved was 96.05% which was from the Self-ResAttentioNet18_Q1 model. Additionally, the five-fold accuracy variance is also the minimum among the other models. The five-fold lowest accuracy 92.97% is achieved by Self-ResAttentioNet18_Q5. From Figure 7, it is evident that Self-ResAttentioNet18_Q1 achieved nearly the same accuracy across the five folds.

The other performance metrics used in this study are overall accuracy, weighted precision, weighted recall, weighted f1 score, and weighted specificity. The performance metrics are recorded in Table 4 for the six different configurations of the two model architectures. The efficiency in classifying the healthy subjects’ signals and ICU patients’ signals was evaluated for q-values of 1, 3, and 5. For the base Self-ResNet18 models, the Self-ResNet18_Q3 model shows superior performance among the three variations. This model achieved an accuracy of 94.91%, 94.92% precision, 94.91% f1 score, and 94.88% specificity. The experiments also were carried out by adding attention layers in the Self-ResNet18 model with the expectation that the overall accuracy would increase significantly. However, the accuracy achieved for the Self-ResAttentioNet18_Q1 model was 2.74% higher than the Self-ResNet18 models. This model achieved an accuracy of 96.05%, 96.06% precision, 96.06% f1 score, and a specificity of 96.09%. Based on the evaluation metrics such as overall accuracy, precision, recall, f1 score, and specificity (as shown in Table 4), Self-ResAttentioNet18 Q1 stands out as the top-performing model among the six variants of Self-ResNet18 and Self-ResAttentioNet18.

Learning curves of a deep learning model, especially the accuracy curves and loss curves, indicate how well the model is performing during training, validation, and testing. The accuracy curve plots the accuracy of the model for training, validation, and test data, while the loss curve plots the loss of the model for training, validation, and test data. The loss curve illustrates the model’s ability to minimize the gap between the predicted and the actual outputs, whilst the accuracy curve demonstrates the model’s ability in learning the features of the data. Both can indicate whether or not the model exhibits overfitting or underfitting. For our models, the top two best-performing variants are Self-ResAttentioNet18_Q1 and Self-ResNet18_Q3.

**Figure 8:**
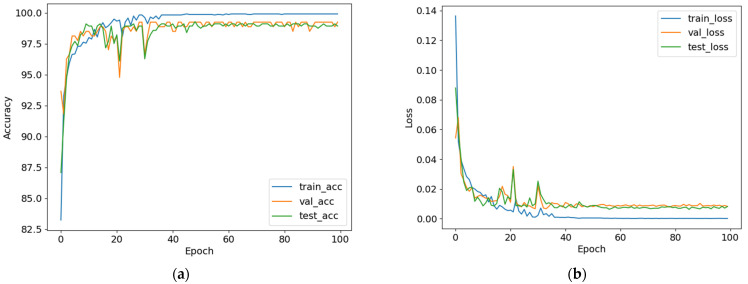
Learning curves of Self-ResAttentionResNet18_Q1. The left graphical representation is the (**a**) accuracy curve and the right graphical representation is the (**b**) loss curve.

Figure 8 shows the learning curves of the Self-ResAttentioNet18_Q1 model, which achieved the highest accuracy for fold 2, among all five folds. The nature of the learning curves shows that the model was not overfitting or underfitting since the training accuracy reaches saturation after gradually improving up to a certain number of epochs. The absence of plateaus in training and validation accuracy points to a deep learning model that is well-fitted. Similar trends can be seen from all the other learning curves presented in Supplementary Tables S1–S6. After analyzing the learning curves for all the variants of our model, Self-ResAttentioNet18_Q1 was found to be the best performing model for session-independent classification of middle cerebral artery doppler ultrasound classification.

### 3.2. ROC Curve and Confusion Matrix-Based Comparison

**Figure 9:**
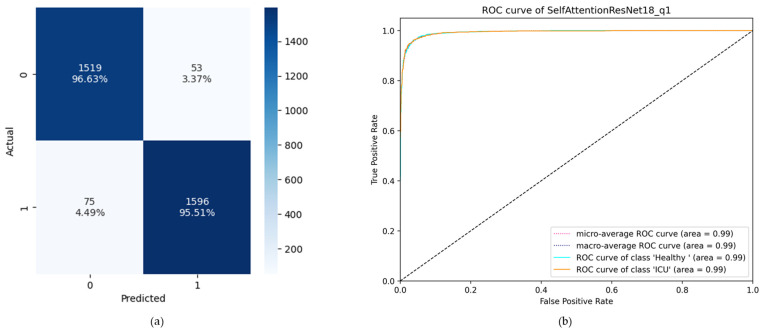
(**a**) Confusion matrix where 0 indicates the Healthy class and 1 indicates the ICU class, and (**b**) ROC curve of Self-ResAttentioNet18_Q1 model.

The receiver operator characteristic (ROC) curve is an important metric for comparing the performance of different binary classification models. The area under the ROC curve (AUC) is a common way to measure how well a classifier does the classification. While a confusion matrix presents corresponding instances of all four TP, TN, FP, and FN, the ROC curve graphically represents the true positive rate against the false positive rate, which can be calculated from the instances of the confusion matrix. From the accuracy-based comparison, it was found that the Self-ResAttentioNet18_Q1 was the best performing model with 96.05% accuracy. The confusion matrix and the ROC curve of this model are presented in Figure 9. The confusion matrix and the ROC curve of the other models can be found in Supplementary Figures S1–S12. The figures show that compared to other models, Self-ResAttentioNet18_Q1 performs better for the classification of healthy subjects and ICU subjects based on the maximal blood flow velocity waveform. The model correctly predicted 95.51% of all the ICU instances with an area of 0.99 under the ROC curve.

### 3.3. Comparison with Respect to Previous Literature

To evaluate the performance of our model, we need to compare it with the existing literature. However, the dataset used in this study has no other associated literature that worked on signal classification. Therefore, the previous literature that worked on a similar problem was used for the comparison. From Table 5, the previous best performing model reported by [45] achieved 89.17% classification accuracy. However, the model proposed in this study surpasses the previous best performing model by achieving a classification accuracy of 96.05% with recall and specificity of 96.05% and 96.09%, respectively.

## 4. Conclusions

In the classification of biomedical signals, deep learning models have been shown to perform exceptionally well. The prognosis and diagnosis of diseases are greatly aided by this type of study. A deep learning model trained on the transcranial doppler (TCD) ultrasound signal, or more specifically MCA waveform, was proposed to be utilized in a binary classification system in this research. Self-ResNet18 and Self-ResAttentioNet18, both based on the SelfONN and ResNet architecture, were proposed in this research. Each deep learning application on biomedical signals needs rigorous testing across many evaluation matrices to guarantee its success. Among the six model versions, Self-ResAttentioNet18_Q1 had the highest classification accuracy at 96.05%, along with the highest recall (96.05%) and the highest specificity (96.09%). A comparative analysis of our proposed model with the existing literature [17,18,45,46] in the normal vs. abnormal classification using ICA or MCA waves has been done in this study to evaluate the performance of Self-AttentioNet18 against contemporary existing models. From that analysis, it can be concluded that the accuracy of the Self-AttentioNet18 in classifying healthy subjects and traumatic brain injured subjects is 6.88% greater than the existing state-of-the-art result [45]. Since both of these studies focus on ultrasound signals from the MCA, the generalizability of our study is also validated. These findings provide more evidence of Self-ResAttentioNet18 for the effectiveness of classifying MCA waveform in the ‘Healthy’ vs. ‘ICU’ classification task. To avoid the data-leakage issue, the entire investigation was planned out using a session-independent technique. This method enables a model to be evaluated using data that were not used during training. The Self-ResAttentioNet18 Q1 performed at an AUC of 0.99 and a classification accuracy of 95.51% when tested on data that had not previously been seen. Such a highly performing deep learning model has the prospect of being used for classifying MCA waveforms in real-time alongside the diagnosis of the targeted patients. This study thus concludes the medical importance of utilizing a Self-ONN-based classification model to classify TCD ultrasonography signals into ‘Healthy’ and ‘ICU’ classifications.

## 5. Limitations and Future Work

Although this study has the potential to detect neurological diseases in real-time, a larger, more diverse dataset can enhance the robustness and reliability of the outcomes. Further studies can be conducted on external datasets as well to assess the generalizability of the model. While this study considers only the signals from the MCA, as a future prospect of this work, signals from other basal arteries such as the internal carotid artery (ICA) can also be investigated and compared to the outcomes using only the MCA signals. The need for human supervision in manual annotation of signals can be eliminated by developing an automated annotator, which will categorize the signals based on their waveform morphologies. In order to improve the signal quality and hence boost the accuracy of the classifier, we plan to explore the signal reconstruction techniques by noise reduction with the use of the proposed model. The use of the proposed Self-AttentioNet18 model can also be explored in the domains of object detection [47] and segmentation [48]. While this study presents promising results and demonstrates the effectiveness of the proposed deep learning models for the classification of TCD ultrasound signals in neurological diseases, it also highlights avenues for further improvement and exploration.

## Figures and Tables

**Table 1:** Overview of the dataset used in this study.

	Healthy	ICU
Number of subjects	6	12
Diagnosis	Healthy	Intraparenchymal hemorrhage, hydrocephalus, and traumatic brain injury (TBI)
Age group	25–45 years	23–74 years
Number of recordings	16	30
The total duration of all recordings	2 h	2 h 40 min
Targeted arteries	MCA	MCA, ICA
Data of targeted artery used in this study	MCA	MCA
Ultrasound system	Philips CX-50 with a 1.75 MHz transducer (S5-1)	
Field of view	90°	
US probe placement	Temporal region and the M1 segment of the MCA	
Blow flow direction for MCA	Towards the probe	
Blood flow velocity for MCA	Positive	

**Table 2:** Fold creation and stratification criteria.

Fold Num	Session ID			
	Train (Healthy)	Test (Healthy)	Train (ICU)	Test (ICU)
1	Rest	“14–16”	Rest	“1, 2, 5”
2	Rest	“10–12”	Rest	“24–26”
3	Rest	“7–10”	Rest	“21–23”
4	Rest	“4–6”	Rest	“4, 5, 20”
5	Rest	“1–3”	Rest	“27–29”

**Table 3:** Training parameters used for binary classification.

Training Parameters	Value
Number of folds	5
Batch size	4
Number of epochs	100
Learning rate	0.0001
Epoch patience	7
Learning factor	0.2
Loss type	SoftM_MSE
Optimization function	Adam

**Table 4:** Performance metrics for six variants of Self-ResNet18 and Self-ResAttentioNet18 different models.

Model Name	Overall Accuracy	Precision	Recall	F1 Score	Specificity
Self-ResNet18_Q1	93.46	93.48	93.46	93.46	93.39
Self-ResNet18_Q3	94.91	94.92	94.91	94.91	94.88
Self-ResNet18_Q5	93.31	93.32	93.31	93.3	93.26
Self- ResAttentioNet18_Q1	96.05	96.06	96.05	96.06	96.09
Self- ResAttentioNet18_Q3	95.31	95.34	95.31	95.31	95.37
Self- ResAttentioNet18_Q5	92.97	93.01	92.97	92.97	93.03

**Table 5:** Evaluation of the proposed model against the background of previous research. The best results are highlighted in bold texts.

Ref.	Data	Classes	Classification Model	Accuracy	Recall	Specificity
[45]	TCD signal from MCA	Vasospasm & normal	Decision tree	89.17	87.5%	89.74%
[18]	Peripheral pulse wave	Five degrees of stenosis	ANN	88.7%	-	-
[46]	Cerebral ultrasound	Stenosis & normal	SVM	80.8% to 81.9%	70.9% to 73.1%	90.7% to 90.8%
[17]	TCD from basal arteries	Stenosis & non-stenosis	RNN	71.1% to 75.89%	74.6% to 75.53%	71.15% to 74.89%
Ours	TCD signal from MCA	Healthy & ICU	Self- ResAttentioNet18_Q1	**96.05%**	**96.05%**	**96.09%**

## Data Availability

The raw TCD data was acquired from IEEE Dataport from the following link: https://ieee-dataport.org/open-access/transcranial-doppler-ultrasound-database-philips-cx50-ultrasound-system (accessed on 3 April 2023).

## Supplementary Materials

The following supporting information can be downloaded at: https://www.mdpi.com/article/10.3390/diagnostics13122000/s1, Figure S1: Confusion matrix for Self-ResNet18_Q1 model; Figure S2: ROC curve of Self-ResNet18_Q1 model; Figure S3: Confusion matrix for Self-ResNet18_Q3 model; Figure S4: ROC curve of Self-ResNet18_Q3 model; Figure S5: Confusion matrix of Self-ResNet18_Q5 model; Figure S6: ROC curve of Self-ResNet18_Q5 model; Figure S7: Confusion matrix of Self-ResAttentioNet18_Q1 model; Figure S8: ROC curve of Self-ResAttentioNet18_Q1 model; Figure S9: Confusion matrix of Self-ResAttentioNet18_Q3 model; Figure S10: ROC curve of Self-ResAttentioNet18_Q3 model; Figure S11: Confusion matrix of Self-ResAttentioNet18_Q5 model; Figure S12: ROC curve of Self-ResAttentioNet18_Q5 model; Table S1: Fold-wise learning curves of the Self-ResNet18_Q1 model; Table S2: Fold-wise learning curves of the Self-ResNet18_Q3 model; Table S3: Fold-wise learning curves of the Self-ResNet18_Q5 model; Table S4: Fold-wise learning curves of the Self-ResAttentionNet18_Q1 model; Table S5: Fold-wise learning curves of the Self-ResAttentionNet18_Q3 model; Table S6: Fold-wise learning curves of the Self-ResAttentionNet18_Q5 model.
