# Supplementary material for: A Deep Learning Framework for the Detection of Abnormality in Cerebral Blood Flow Velocity Using Transcranial Doppler Ultrasound
Source: Diagnostics (Basel). 2023 Jun 8;13(12):2000. doi: 10.3390/diagnostics13122000 (PMC10297317; doi:10.3390/diagnostics13122000)
Supplement: Supplementary file 1 [file diagnostics-13-02000-s001.zip › diagnostics-2353558-supplementary.pdf]

Supplementary Materials

Self-ResNet18_Q1 Model

Table S1: Fold-wise learning curves of the Self-ResNet18_Q1 model

<table>
<tr><th>Fold</th><th>Accuracy Curve</th><th>Loss Curve</th></tr>
<tr><td>1</td><td>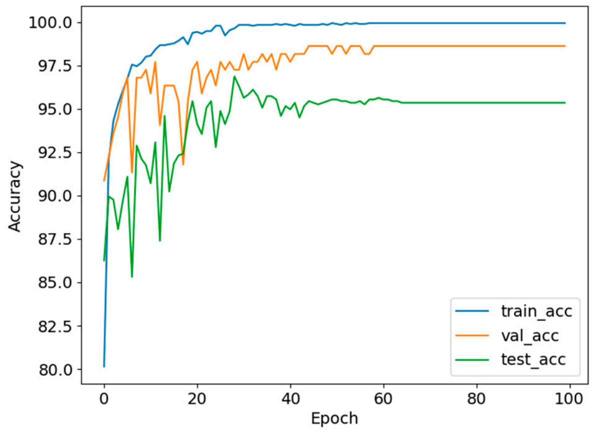
</td><td>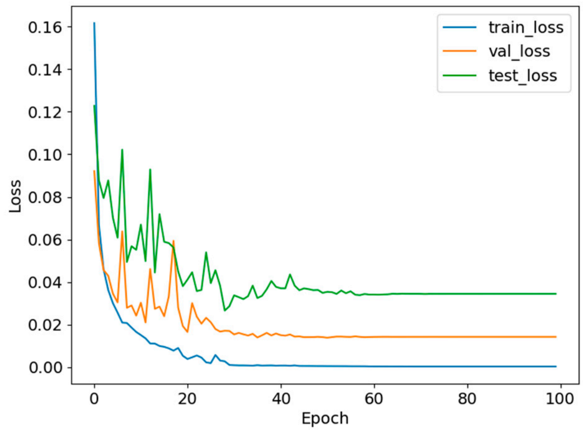
</td></tr>
<tr><td>2</td><td>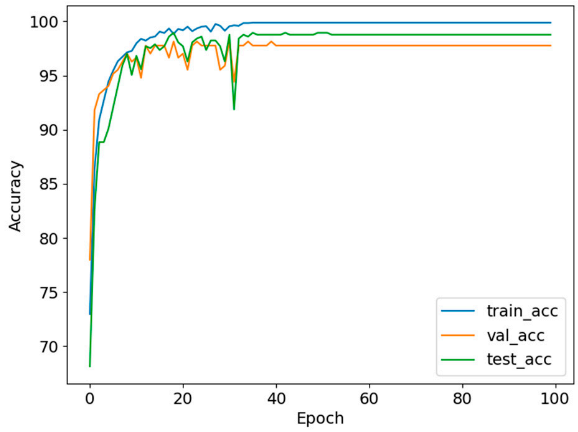
</td><td>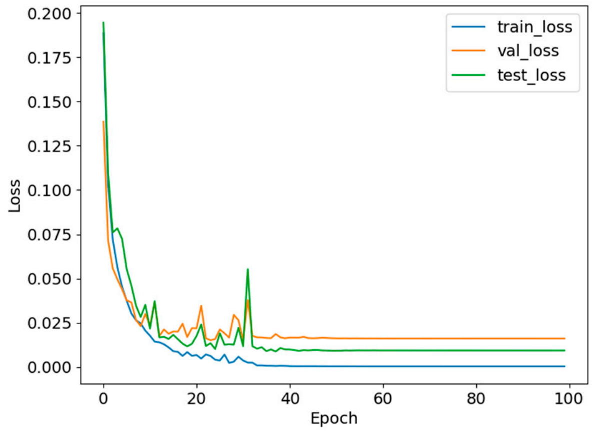
</td></tr>
</table>

3

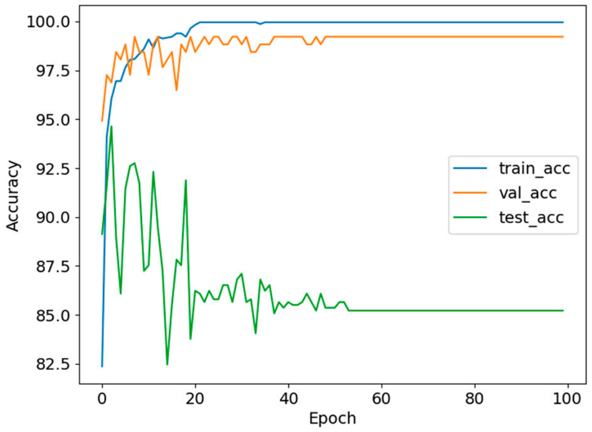

Accuracy
100.0
97.5
95.0
92.5
90.0
87.5
85.0
82.5
0
20
40
60
80
100
Epoch
train_acc
val_acc
test_acc

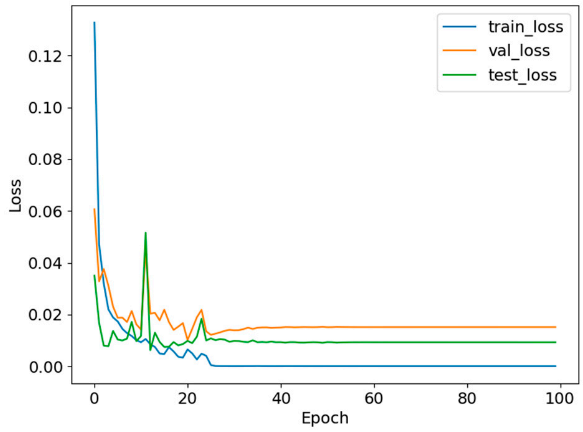

Loss
0.12
0.10
0.08
0.06
0.04
0.02
0.00
0
20
40
60
80
100
Epoch
train_loss
val_loss
test_loss

4

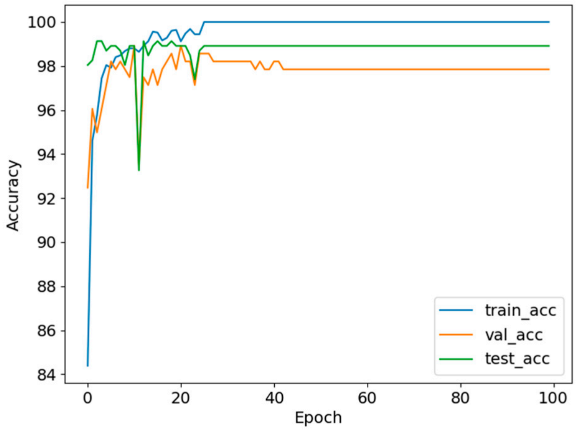

Accuracy
100
98
96
94
92
90
88
86
84
0
20
40
60
80
100
Epoch
train_acc
val_acc
test_acc

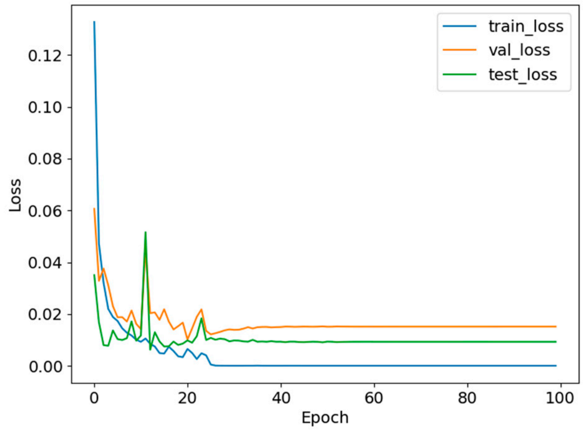

Loss
0.12
0.10
0.08
0.06
0.04
0.02
0.00
0
20
40
60
80
100
Epoch
train_loss
val_loss
test_loss

5

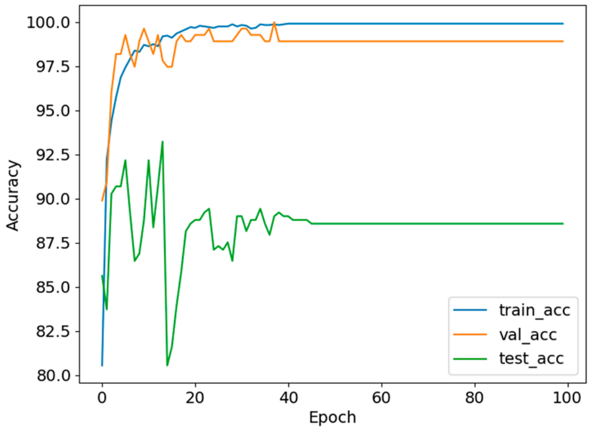

Accuracy
100.0
97.5
95.0
92.5
90.0
87.5
85.0
82.5
80.0
0
20
40
60
80
100
Epoch
train_acc
val_acc
test_acc

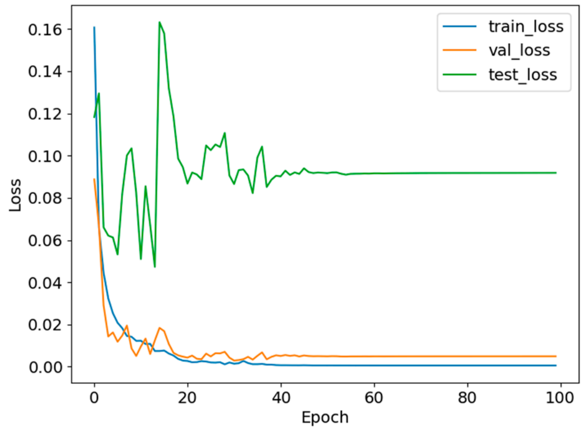

Loss
0.16
0.14
0.12
0.10
0.08
0.06
0.04
0.02
0.00
0
20
40
60
80
100
Epoch
train_loss
val_loss
test_loss

The confusion matrix of Self-ResNet18_Q1 model is given in Figure 1.

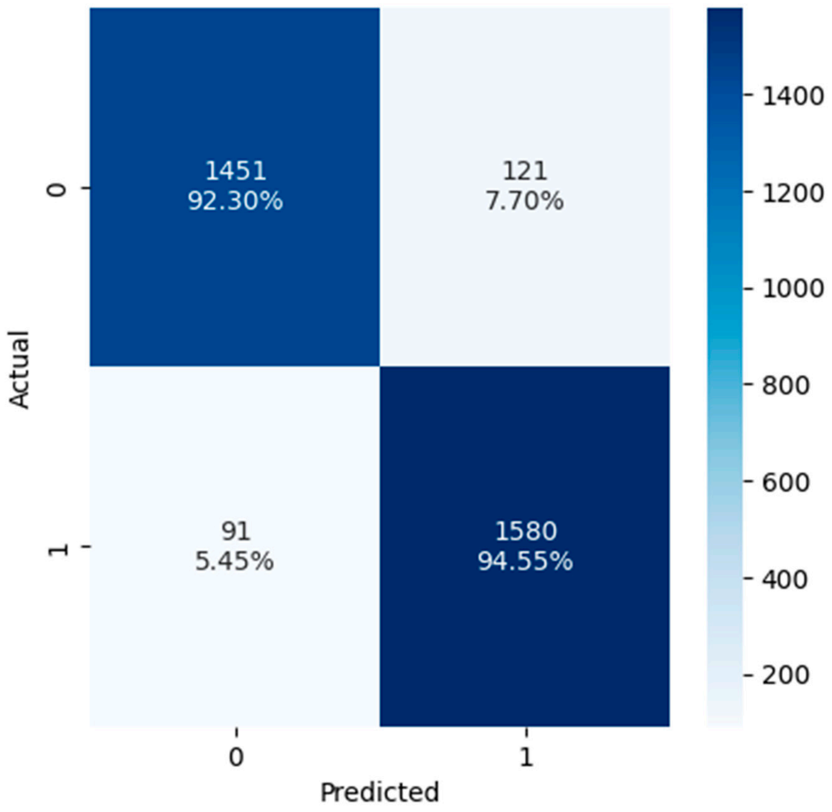

Figure S1: Confusion matrix for Self-ResNet18_Q1 model

The Receiver operator characteristics (ROC) curve of Self-ResNet18_Q1 model is given in Figure 2 below.

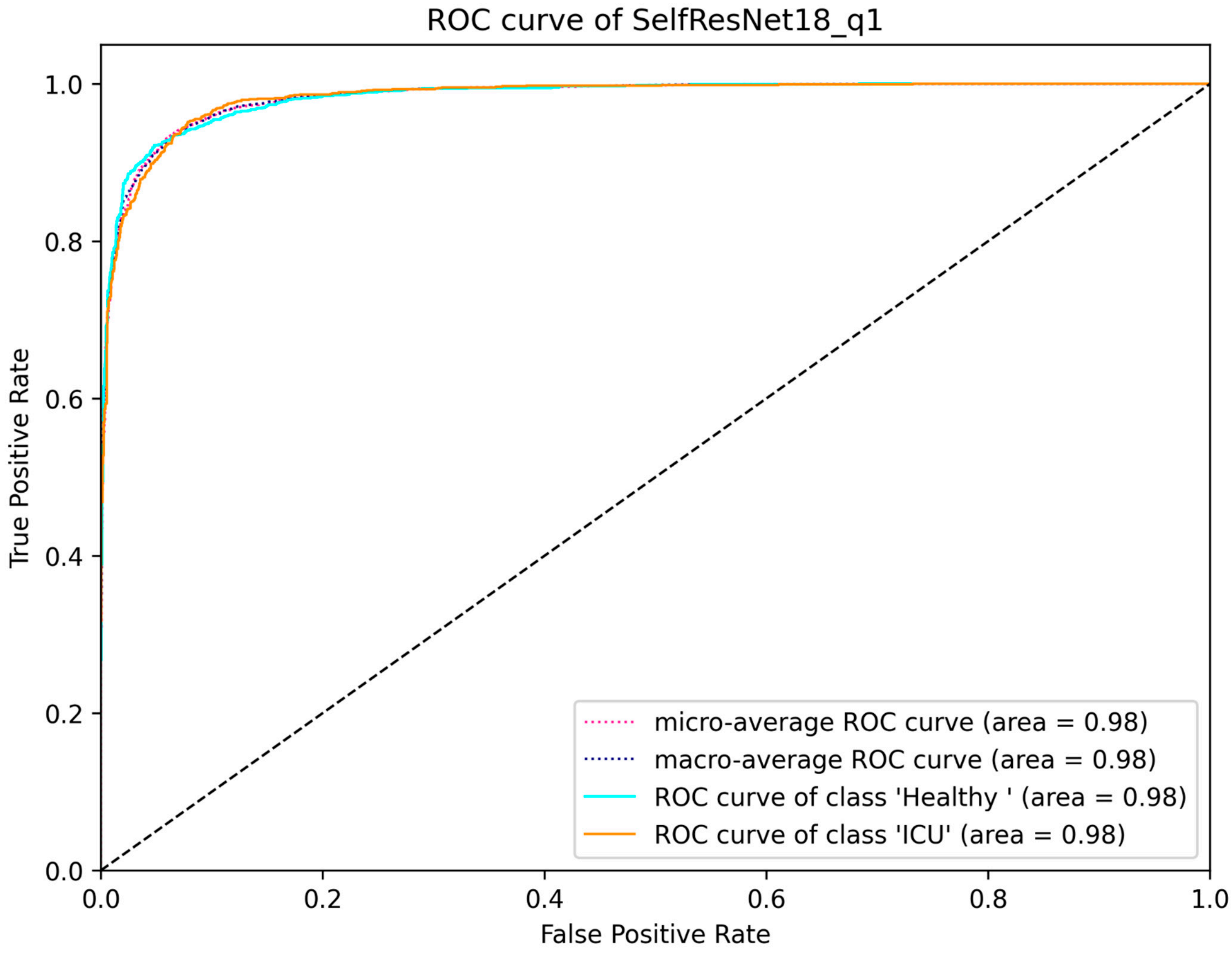

Figure S2: ROC curve of Self-ResNet18_Q1 model

Self-ResNet18_Q3 Model

Table S2: Fold-wise learning curves of the Self-ResNet18_Q3 model

Fold	Accuracy Curve	Loss Curve
1	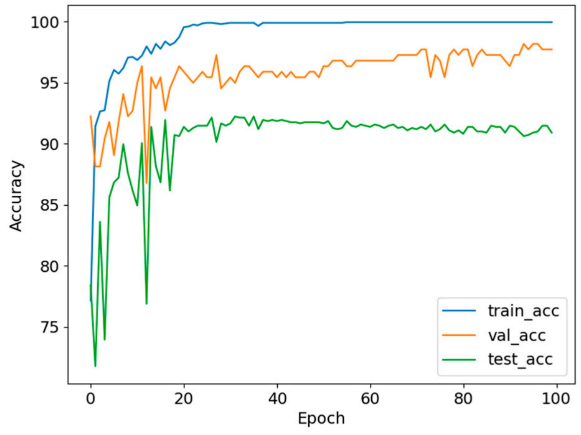	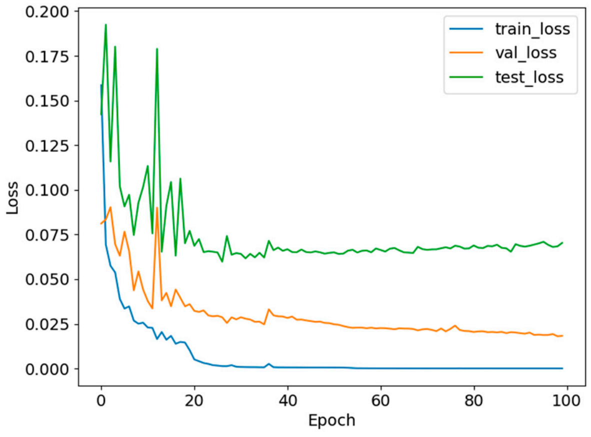
2	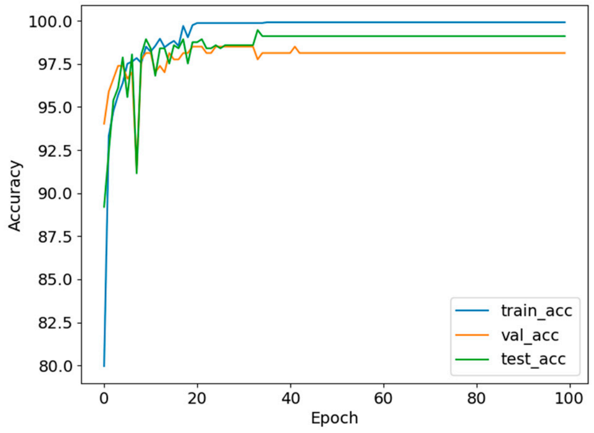	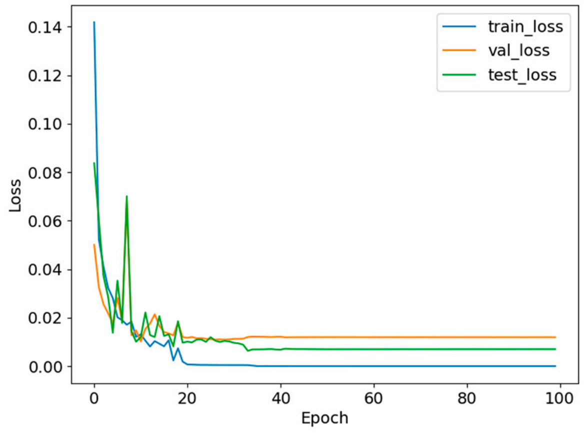
3	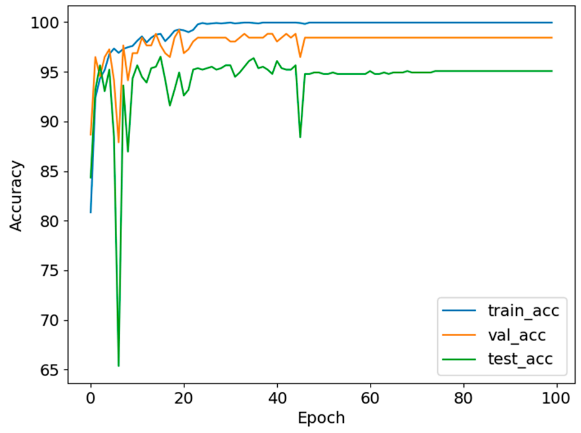	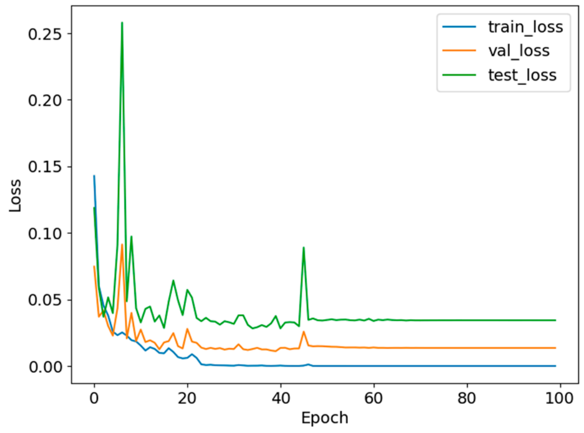

<table>
<tr><td>4</td><td>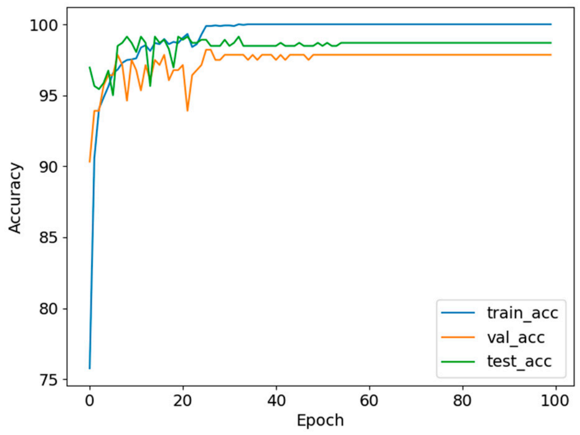
</td><td>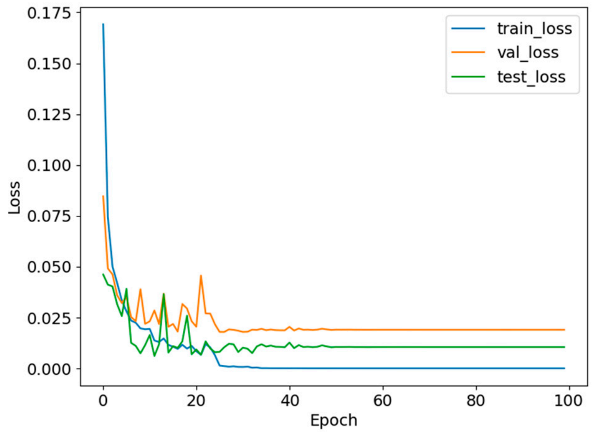
</td></tr>
<tr><td>5</td><td>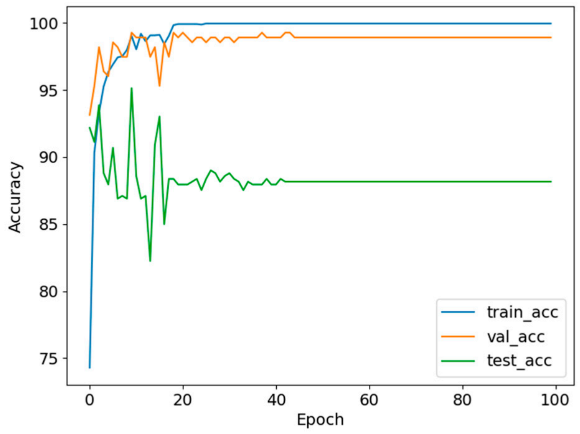
</td><td>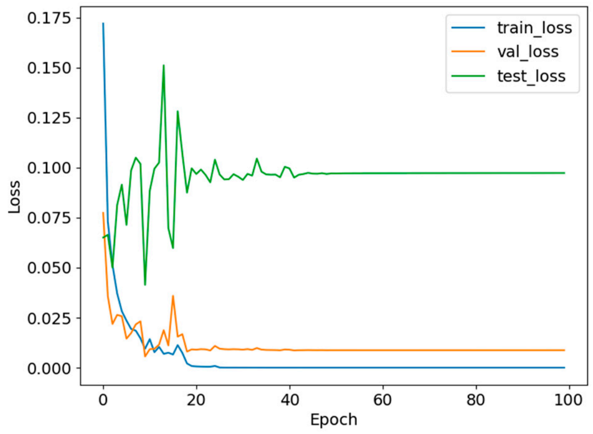
</td></tr>
</table>

The confusion matrix of Self-ResNet18_Q3 model is given in Figure 3.

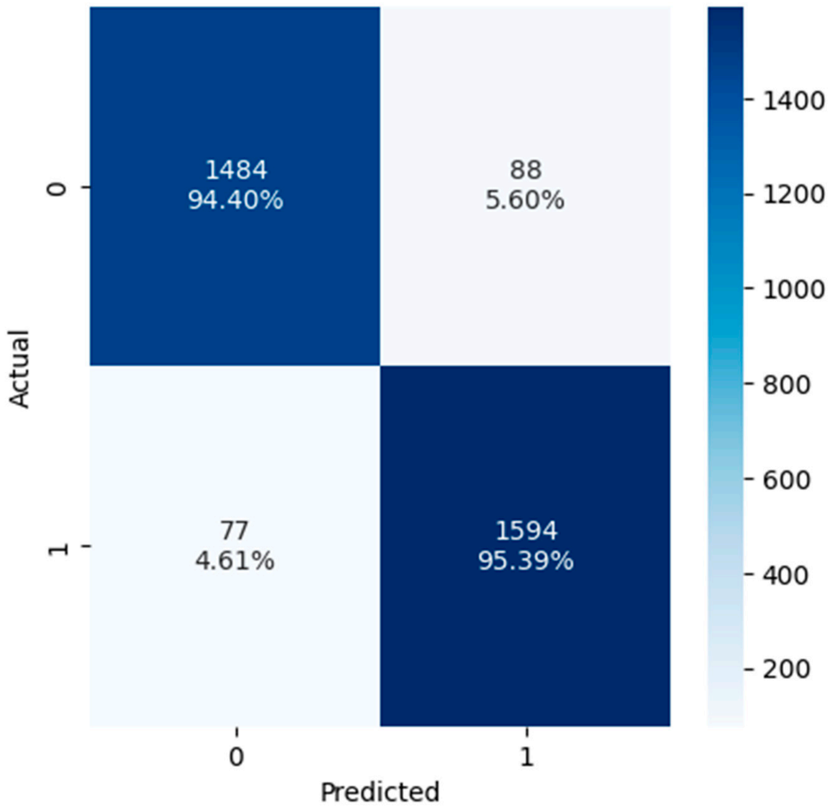

Figure S3: Confusion matrix for Self-ResNet18_Q3 model

The Receiver operator characteristics (ROC) curve of Self-ResNet18_Q3 model is given in Figure 4 below.

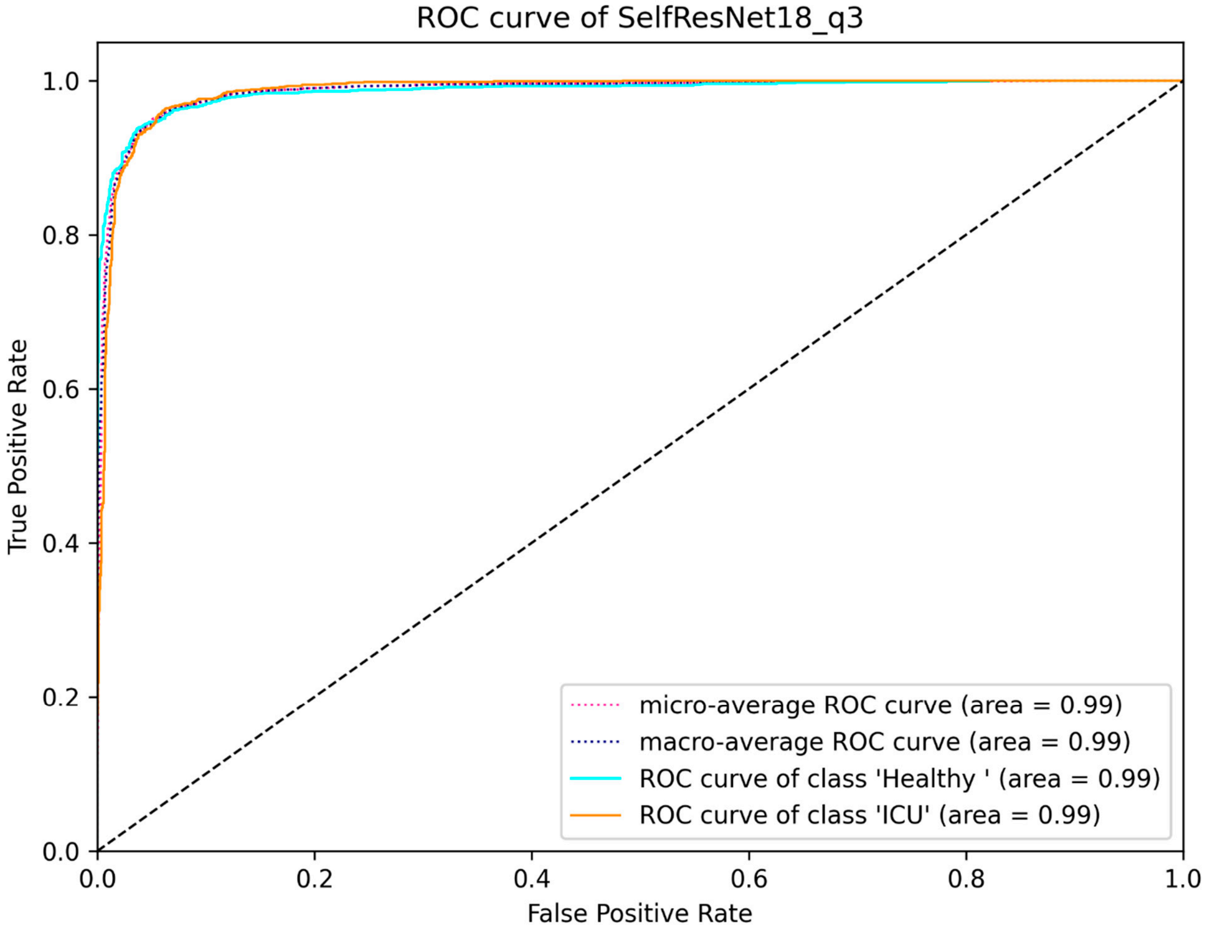

Figure S4: ROC curve of Self-ResNet18_Q3 model

Self-ResNet18_Q5 Model

Table S3: Fold-wise learning curves of the Self-ResNet18_Q5 model

<table>
<tr><th>Fold</th><th>Accuracy Curve</th><th>Loss Curve</th></tr>
<tr><td>1</td><td>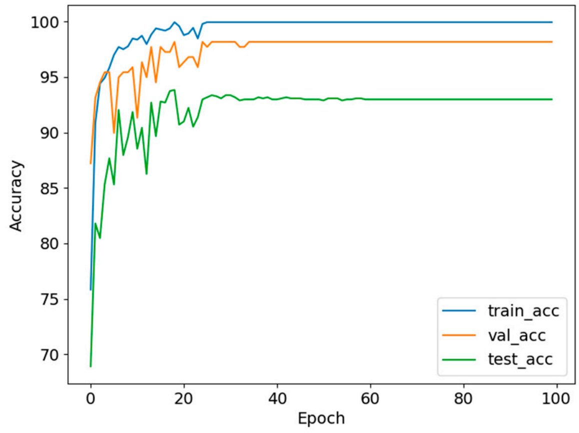
</td><td>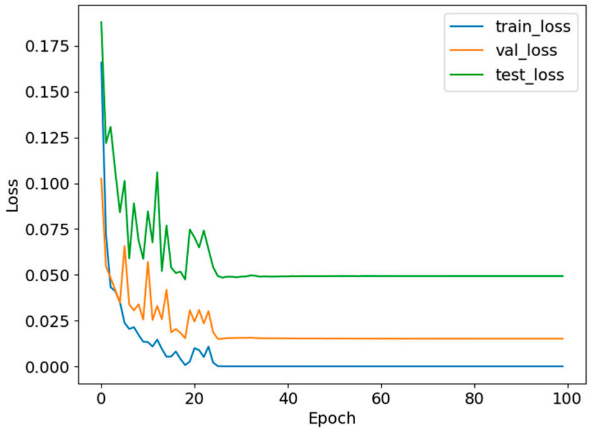
</td></tr>
<tr><td>2</td><td>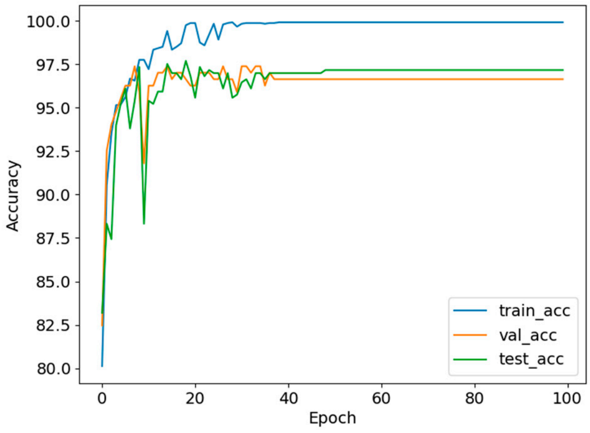
</td><td>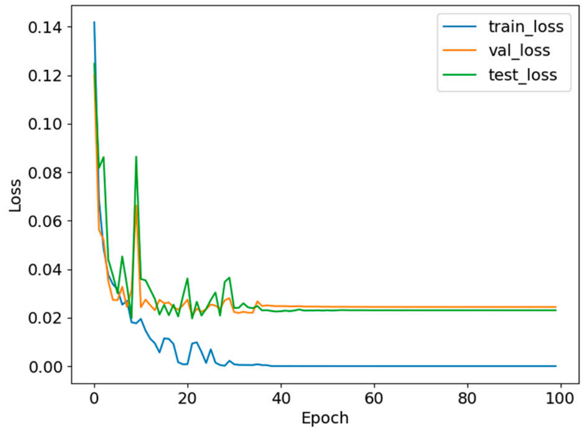
</td></tr>
<tr><td>3</td><td>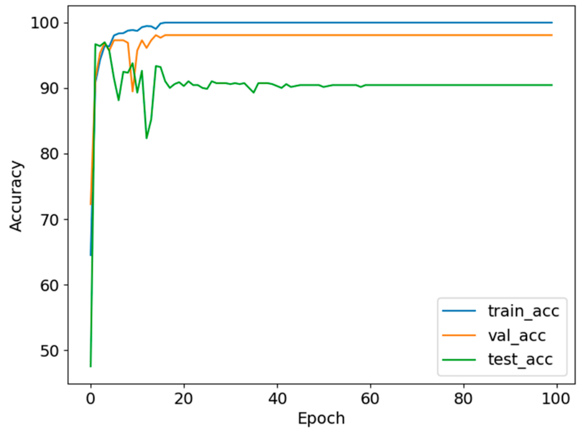
</td><td>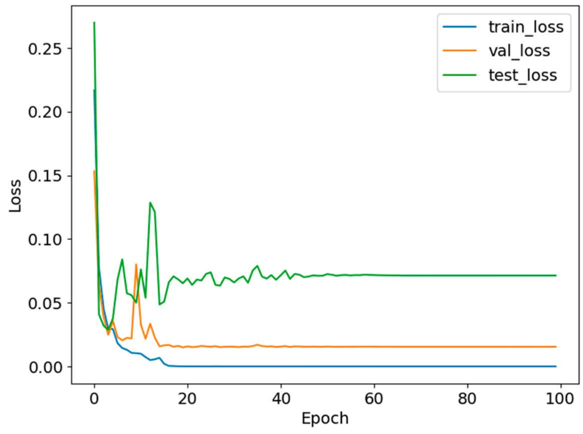
</td></tr>
</table>

<table>
<tr><td>4</td><td>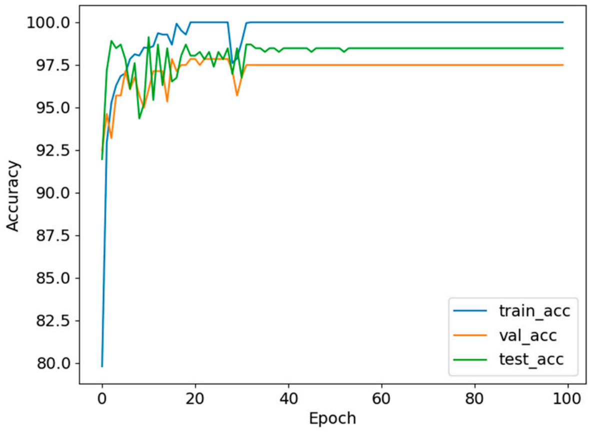
</td><td>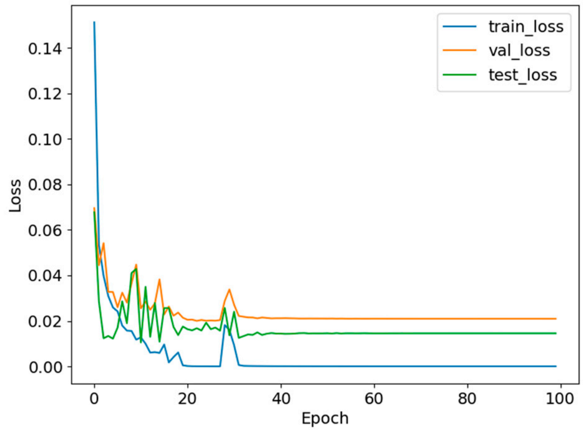
</td></tr>
<tr><td>5</td><td>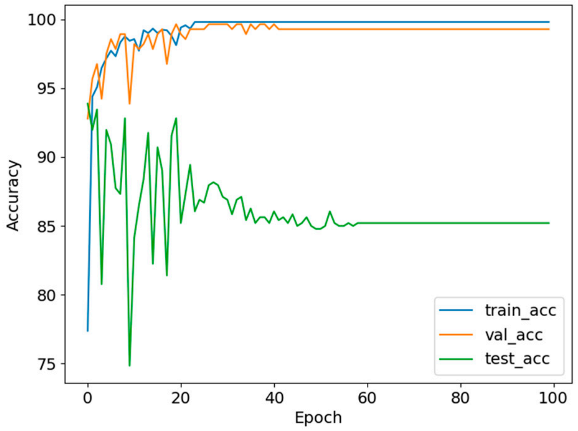
</td><td>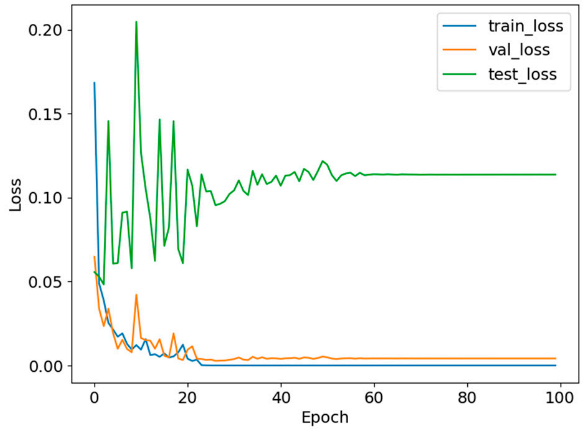
</td></tr>
</table>

The confusion matrix of Self-ResNet18_Q5 model is given in Figure 5.

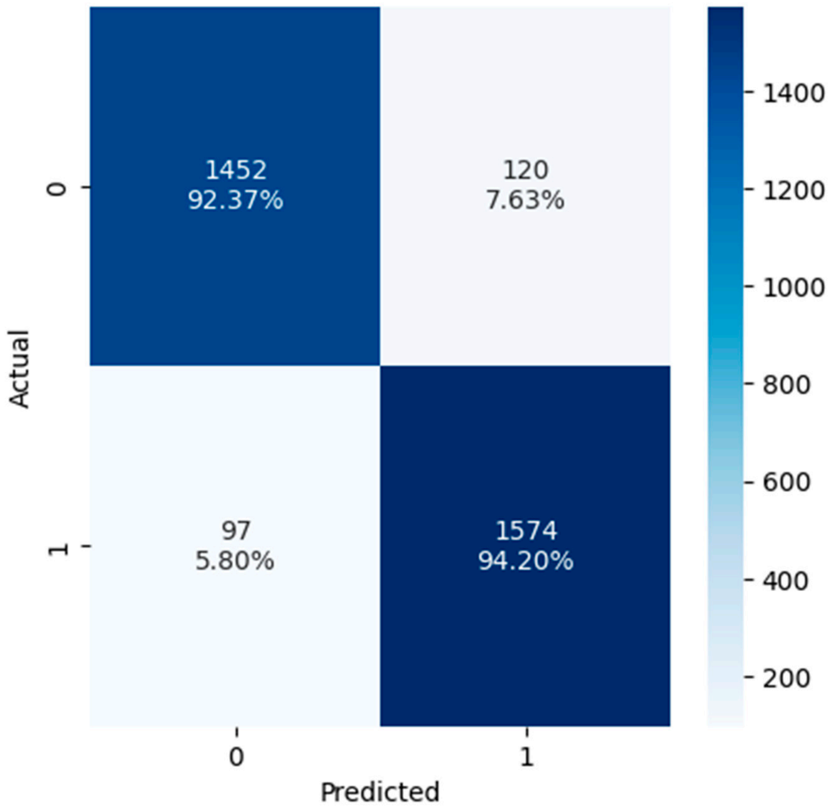

Figure S5: Confusion matrix of Self-ResNet18_Q5 model

The ROC curve of Self-ResNet18_Q5 model is given in Figure 6 below.

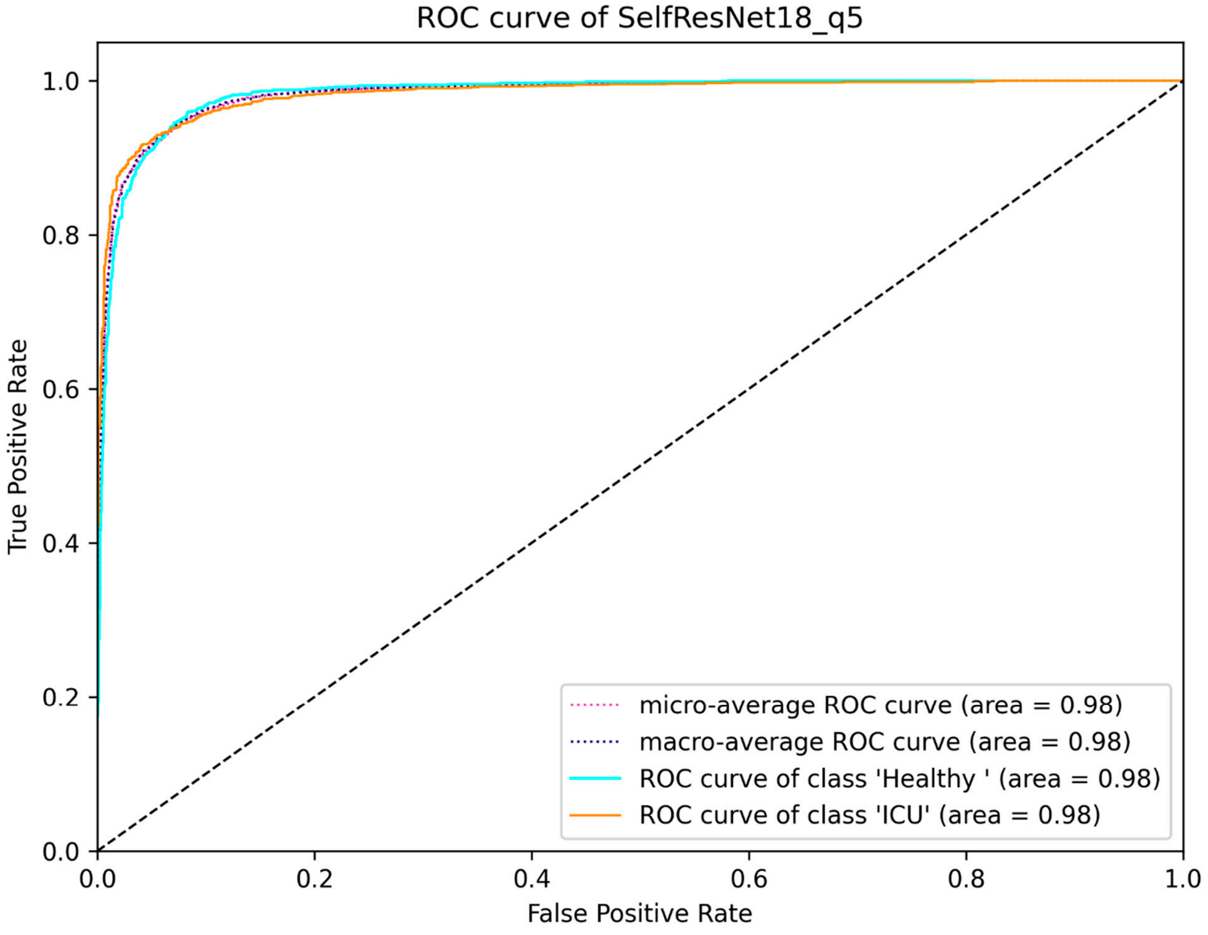

Figure S6: ROC curve of Self-ResNet18_Q5 model

Self-ResAttentioNet18_Q1 Model

Table S4: Fold-wise learning curves of the Self-ResAttentionNet18_Q1 model

<table>
<tr><th>Fold</th><th>Accuracy Curve</th><th>Loss Curve</th></tr>
<tr><td>1</td><td>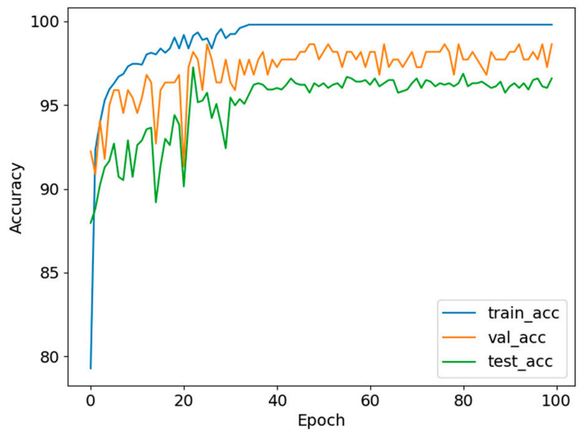
</td><td>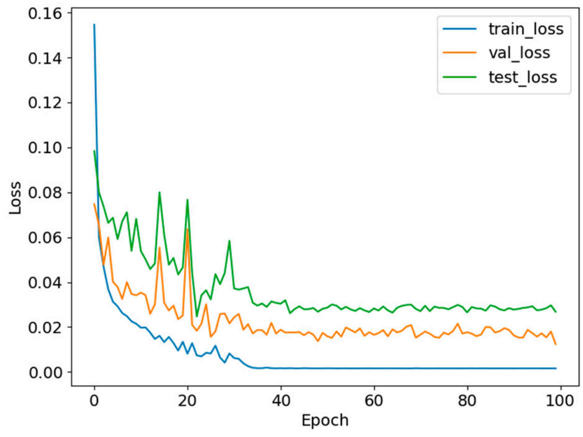
</td></tr>
<tr><td>2</td><td>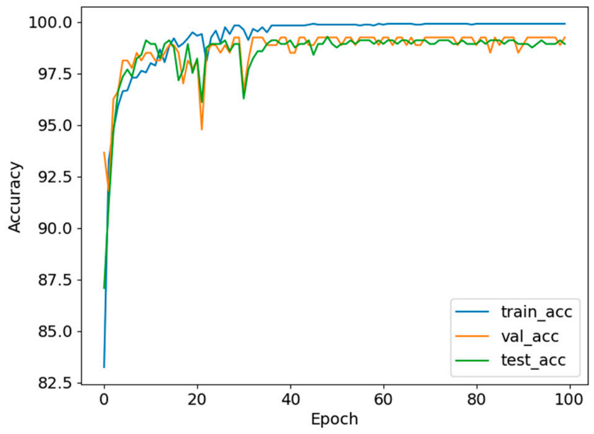
</td><td>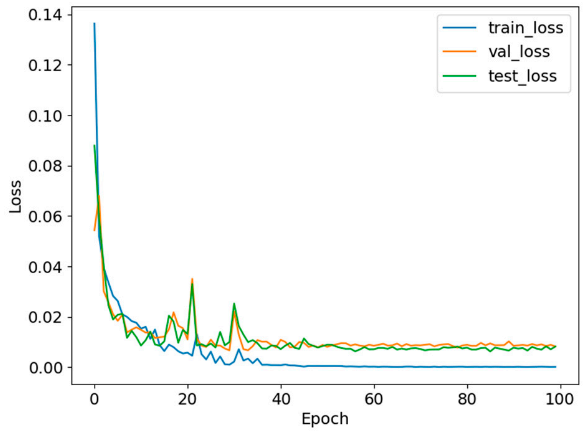
</td></tr>
<tr><td>3</td><td>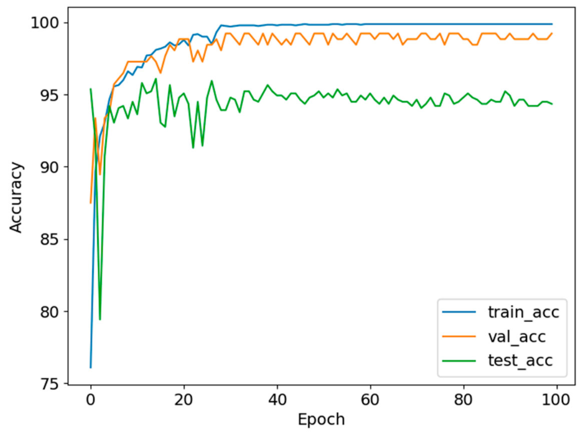
</td><td>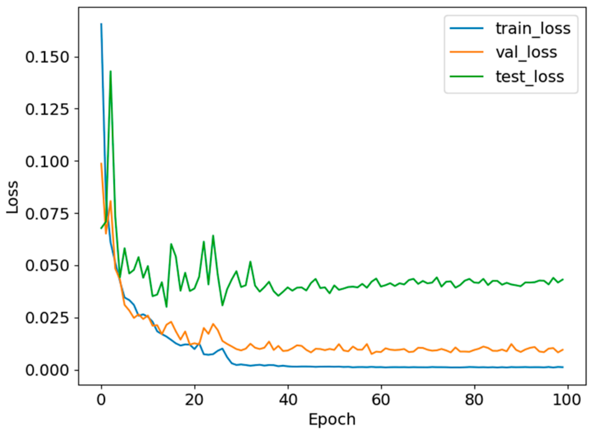
</td></tr>
</table>

<table>
<tr><td>4</td><td>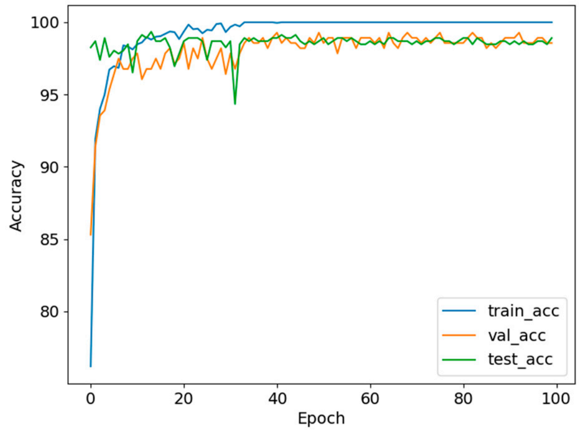
</td><td>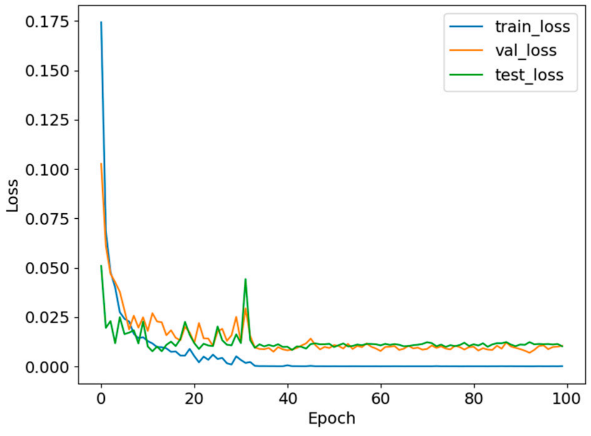
</td></tr>
<tr><td>5</td><td>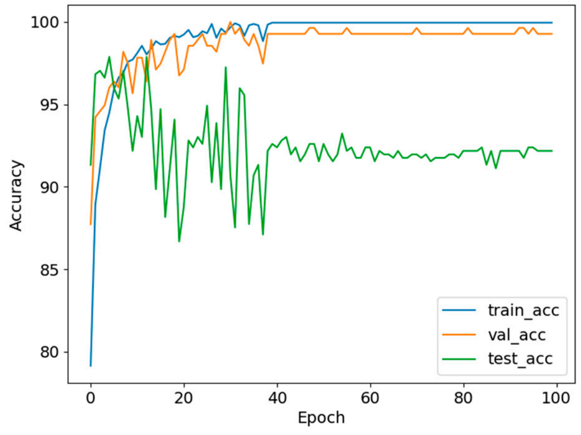
</td><td>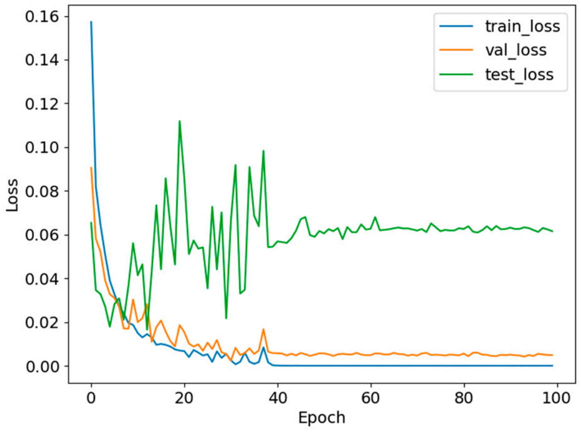
</td></tr>
</table>

The confusion matrix of Self-ResAttentioNet18_Q1 model is given in Figure 7.

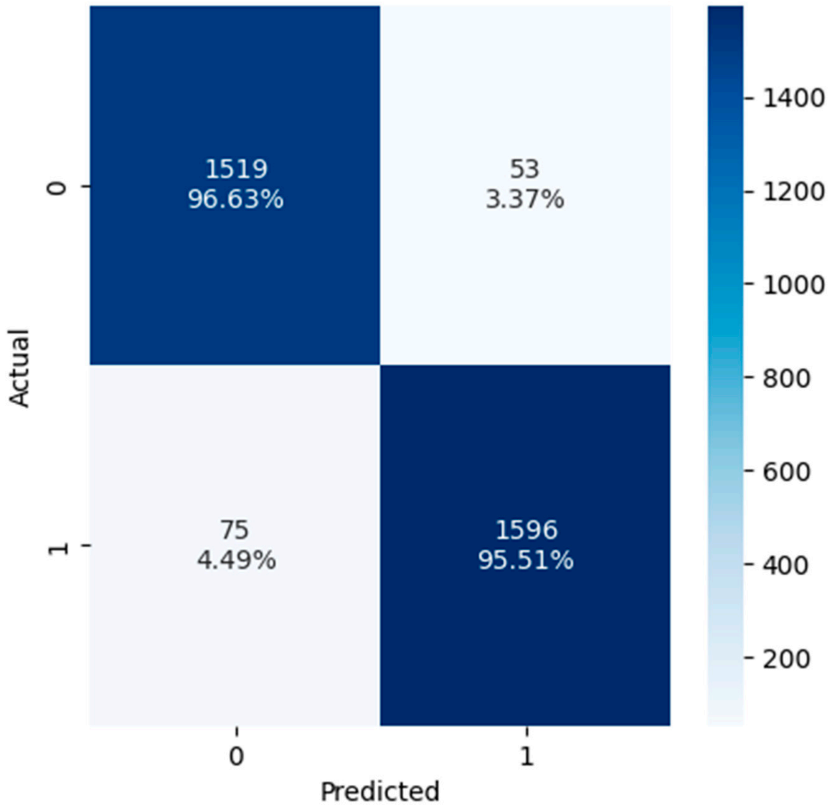

Figure S7: Confusion matrix of Self-ResAttentioNet18_Q1 model

The ROC curve of Self-ResAttentioNet18_Q1 model is given in Figure 8 below.

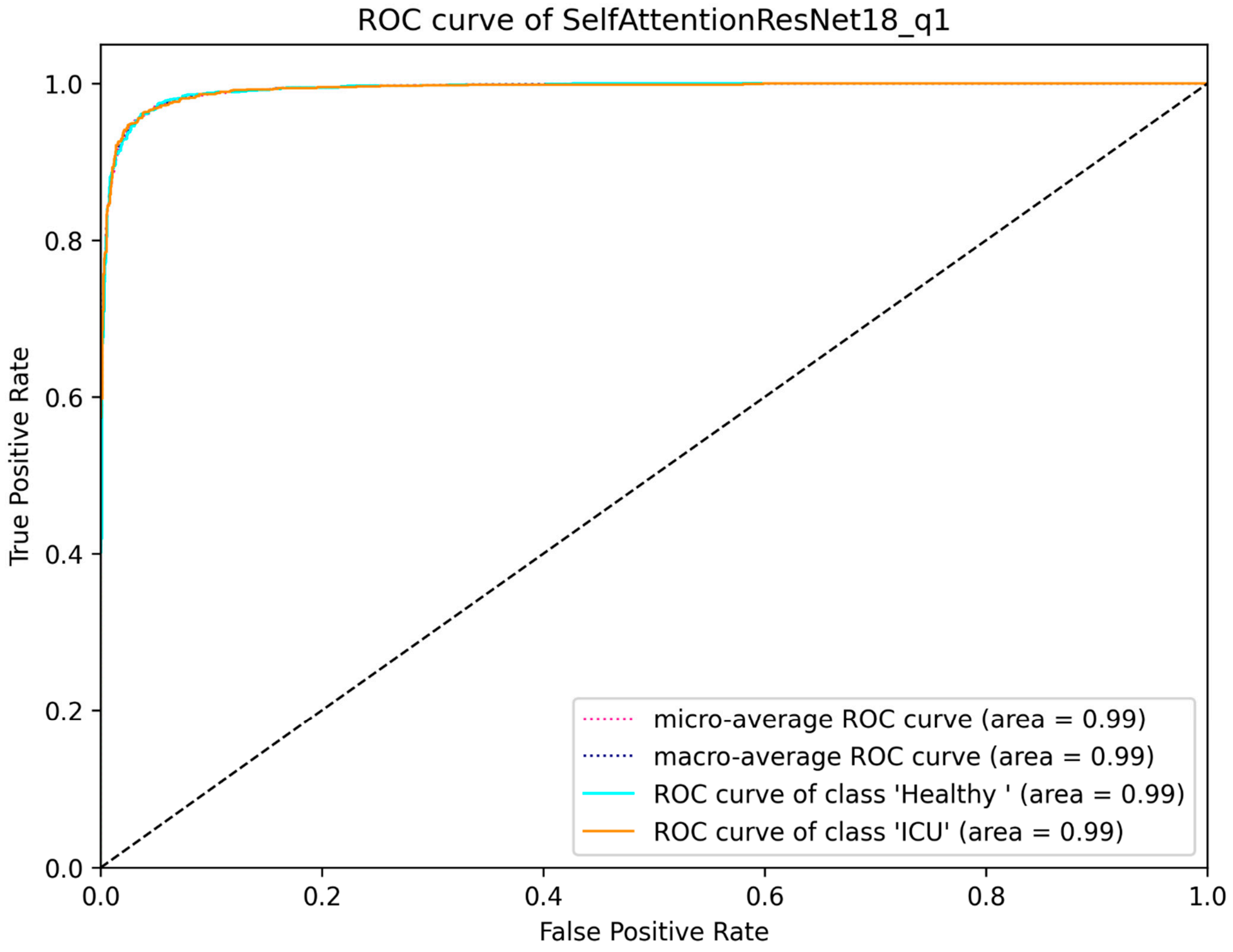

Figure S8: ROC curve of Self-ResAttentioNet18_Q1 model

Self-ResAttentioNet18_Q3 Model

Table S5: Fold-wise learning curves of the Self-ResAttentionNet18_Q3 model

Fold	Accuracy Curve	Loss Curve
1	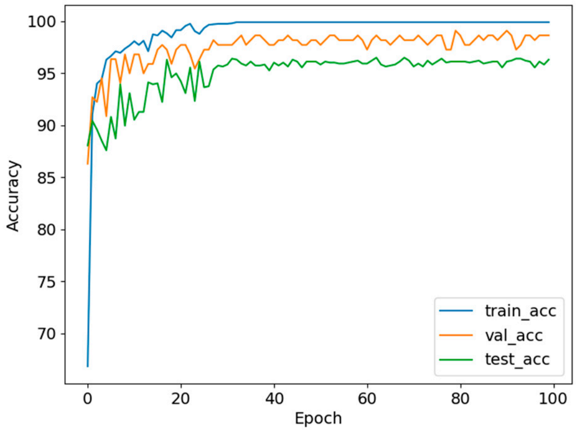	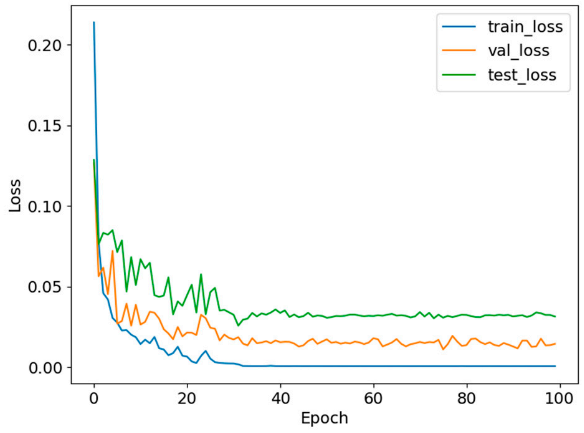
2	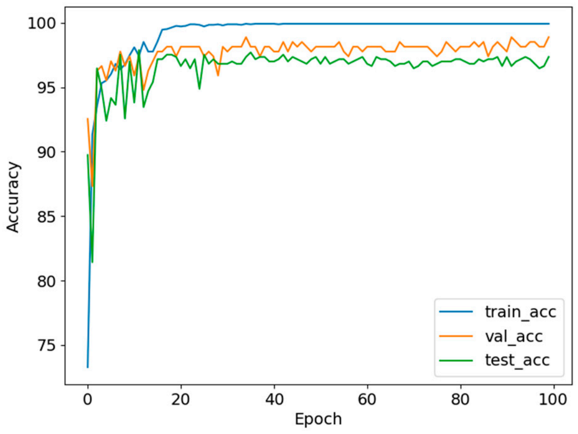	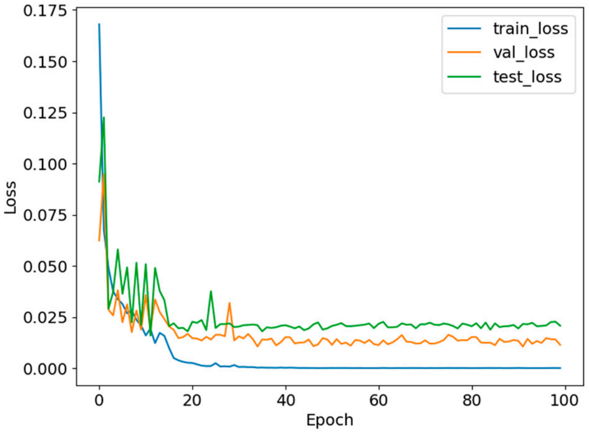
3	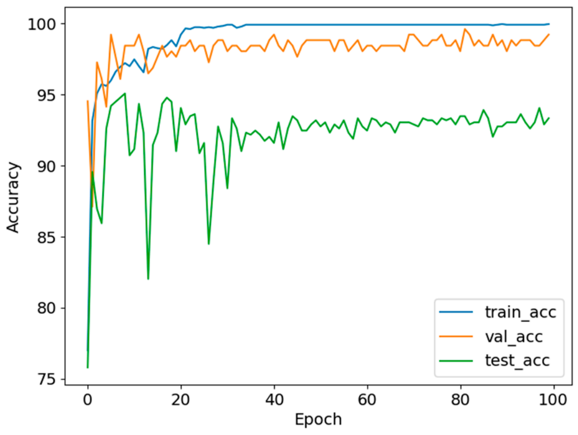	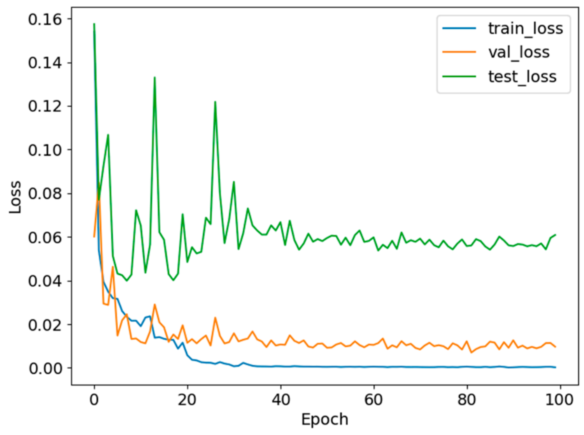

<table>
<tr>
<td>4</td>
<td>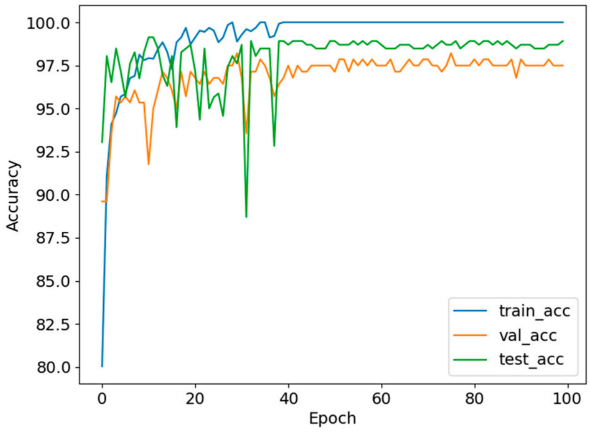
</td>
<td>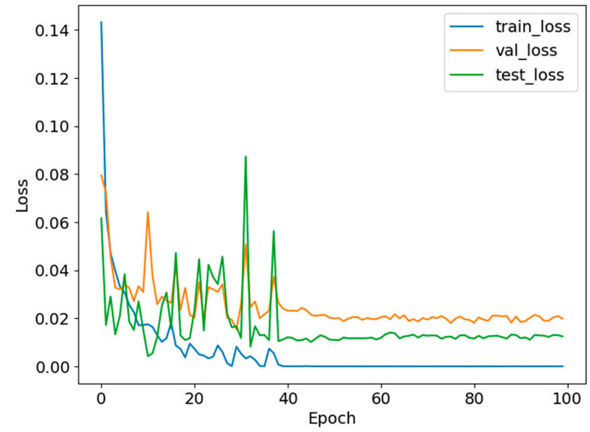
</td>
</tr>
<tr>
<td>5</td>
<td>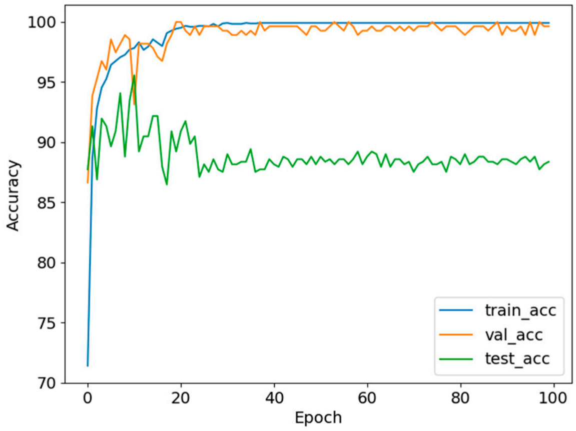
</td>
<td>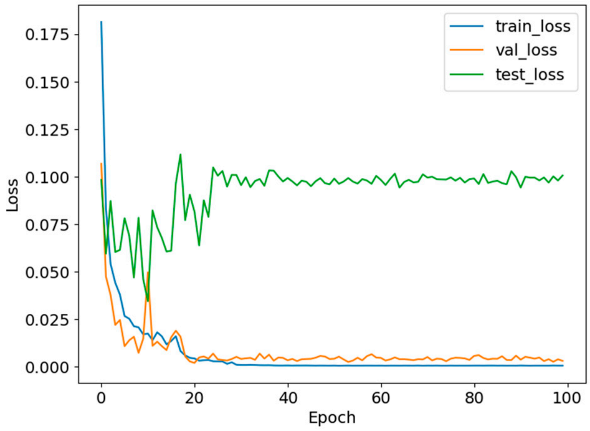
</td>
</tr>
</table>

The confusion matrix of Self-ResAttentioNet18_Q3 model is given in Figure 9.

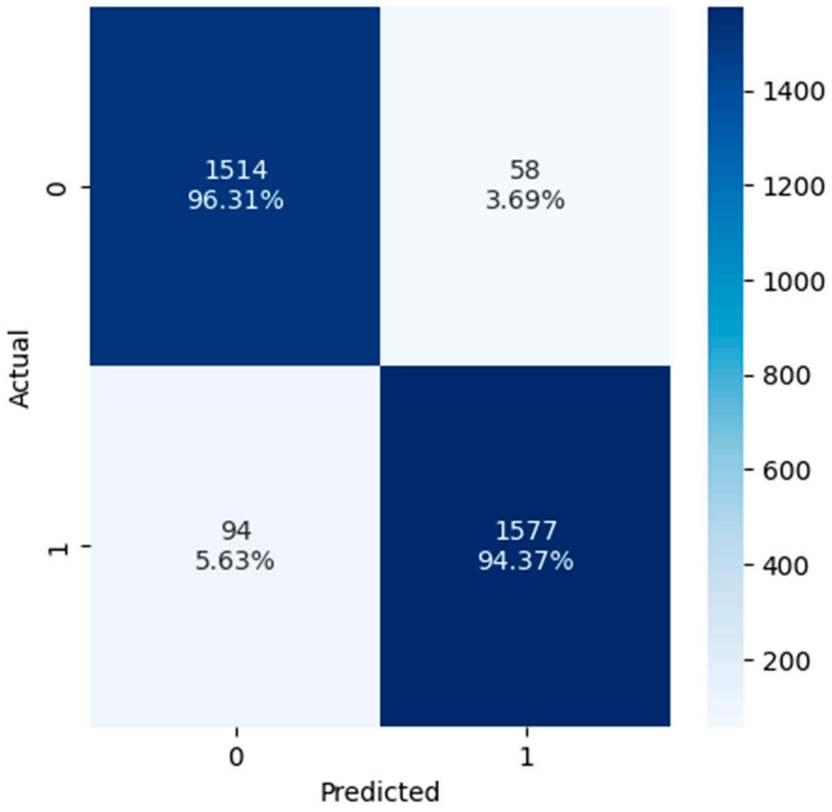

Figure S9: Confusion matrix of Self-ResAttentioNet18_Q3 model

The ROC curve of Self-ResAttentioNet18_Q3 model is given in Figure 10 below.

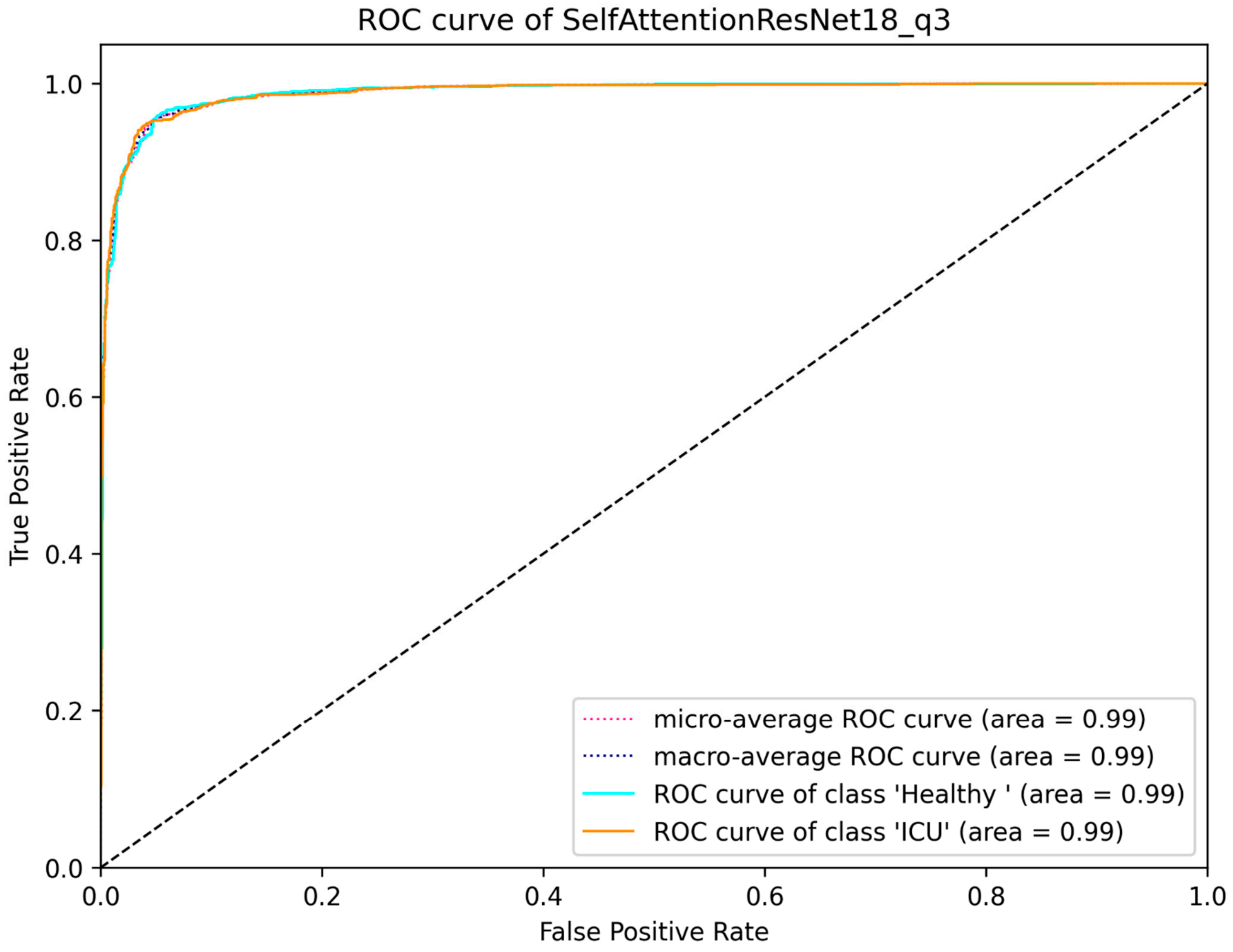

Figure S10: ROC curve of Self-ResAttentioNet18_Q3 model

Self-ResAttentioNet18_Q5 Model

Table S6: Fold-wise learning curves of the Self-ResAttentionNet18_Q5 model

<table>
<tr><th>Fold</th><th>Accuracy Curve</th><th>Loss Curve</th></tr>
<tr><td>1</td><td>
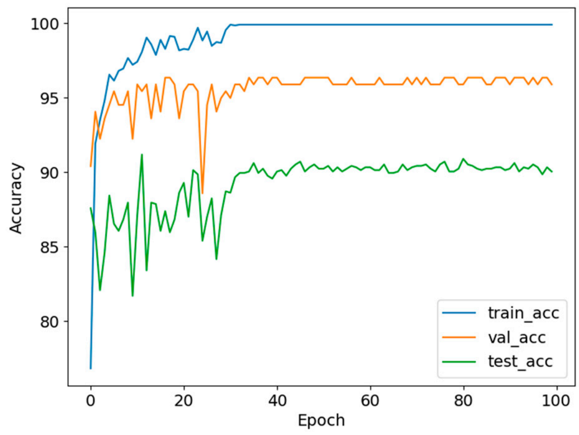

</td><td>
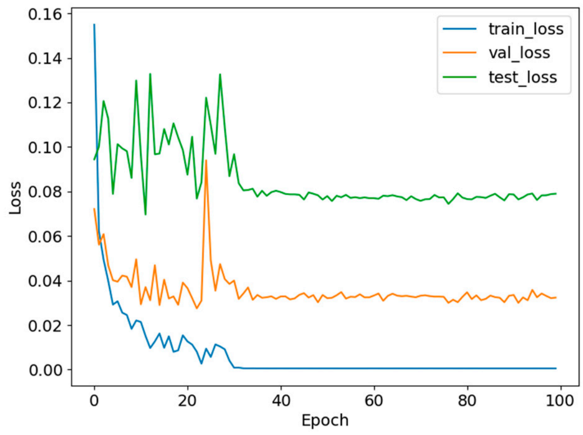

</td></tr>
<tr><td>2</td><td>
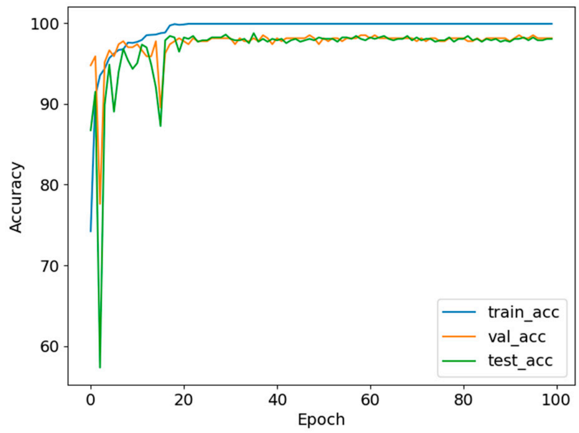

</td><td>
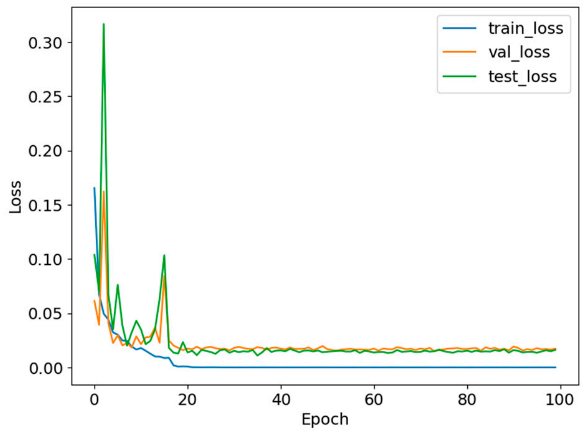

</td></tr>
<tr><td>3</td><td>
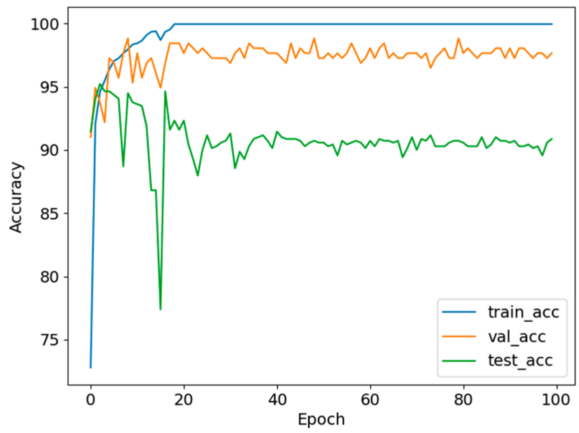

</td><td>
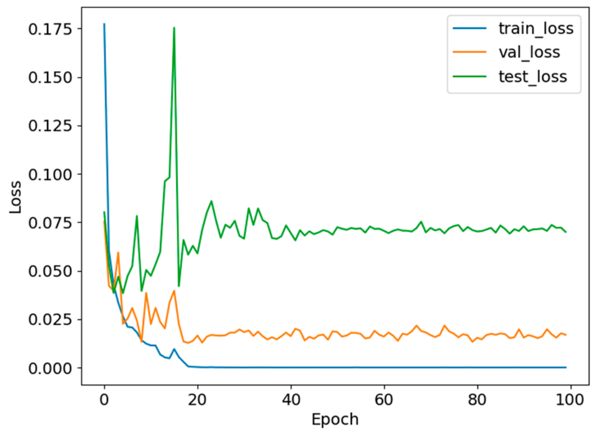

</td></tr>
</table>

<table>
<tr>
<td>4</td>
<td>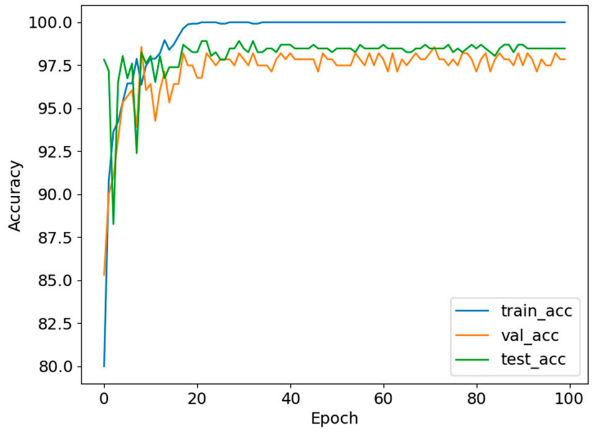
</td>
<td>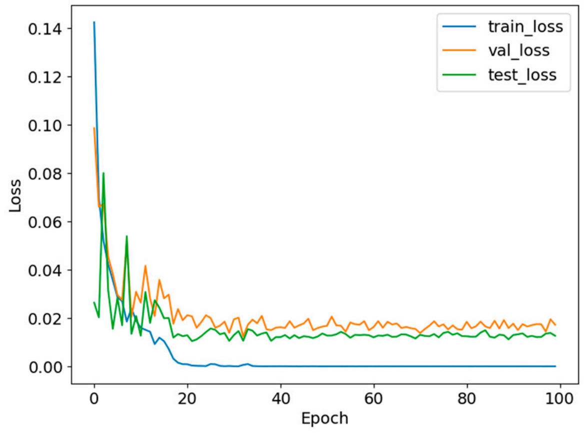
</td>
</tr>
<tr>
<td>5</td>
<td>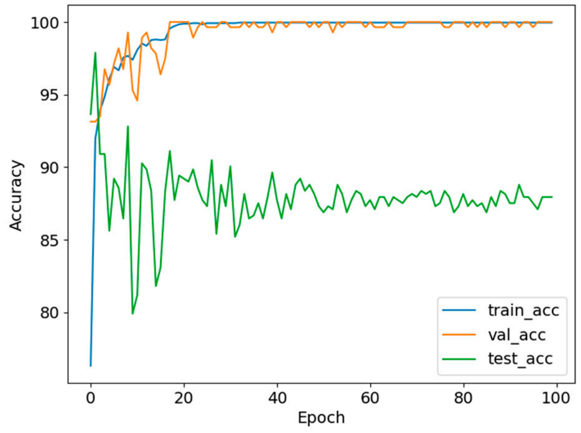
</td>
<td>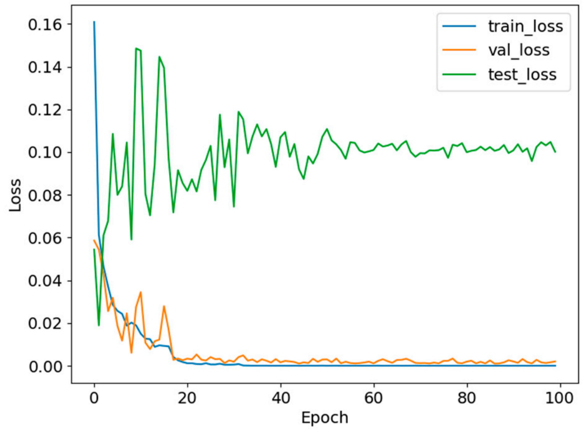
</td>
</tr>
</table>

The confusion matrix of Self-ResAttentioNet18_Q5 model is given in Figure 11.

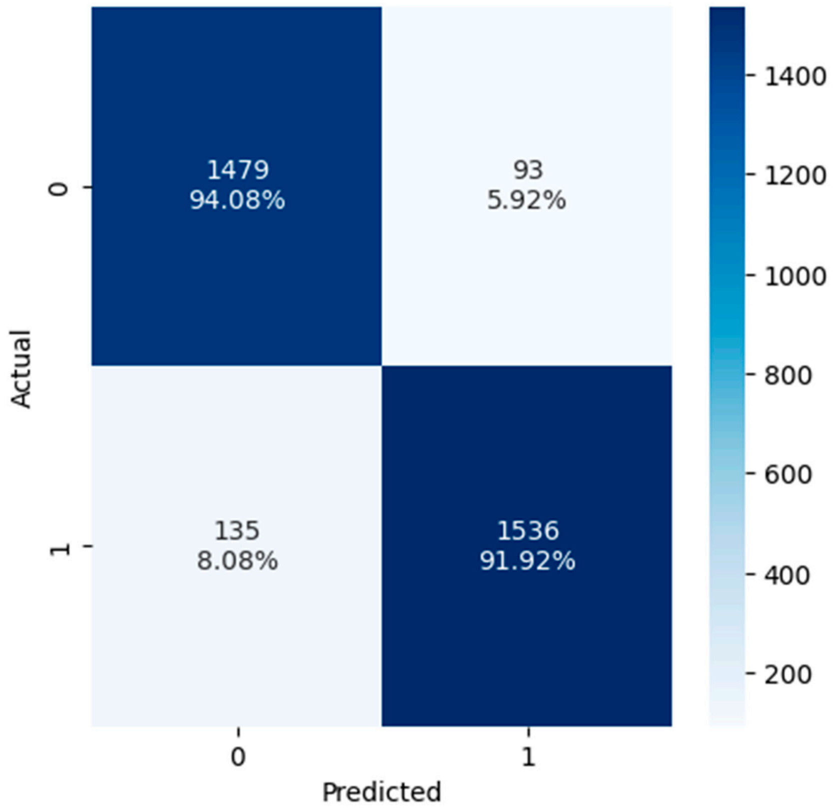

Figure S11: Confusion matrix of Self-ResAttentioNet18_Q5 model

The ROC curve of Self-ResAttentioNet18_Q5 model is given in Figure 12 below.

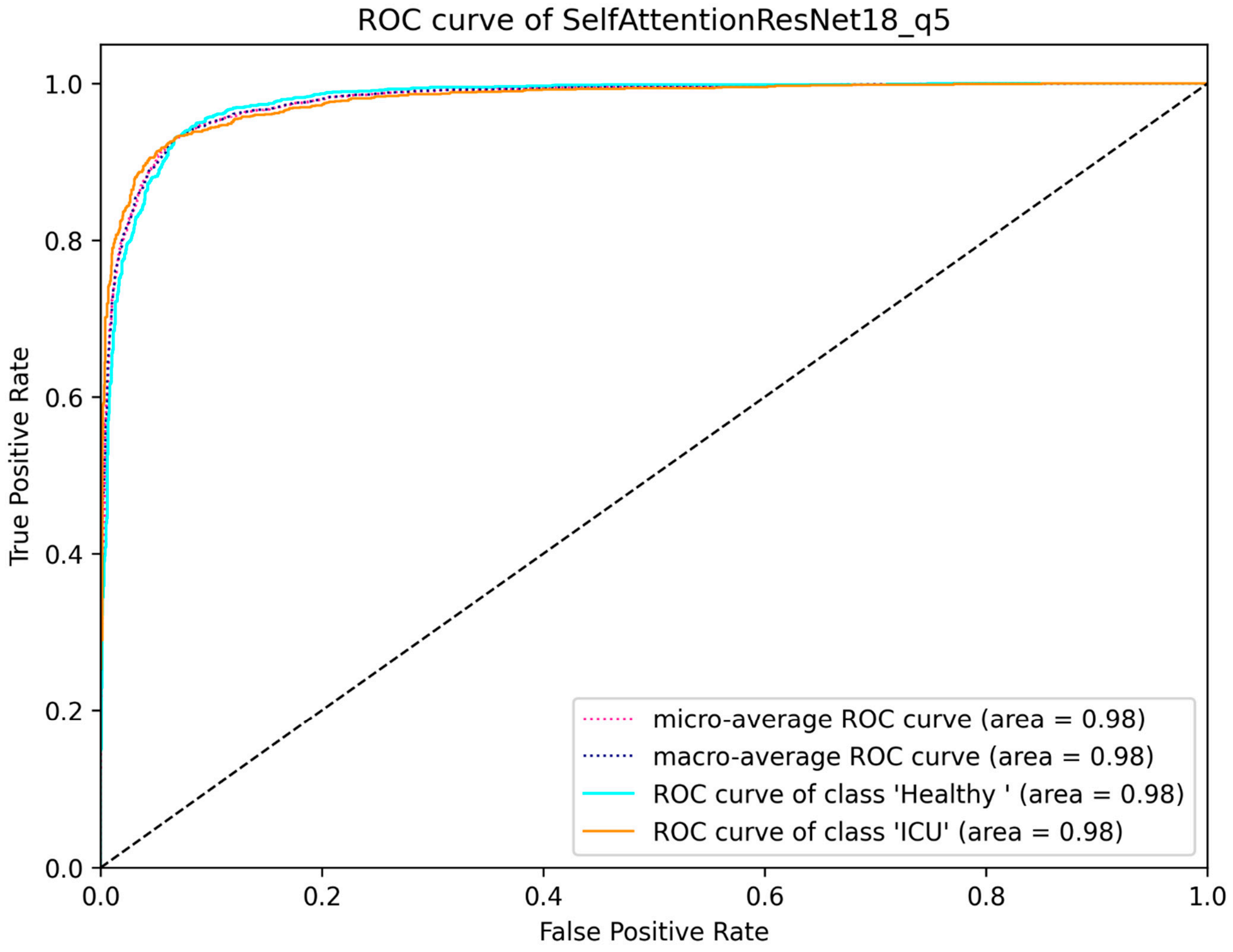

Figure S12: ROC curve of Self-ResAttentioNet18_Q5 model
